# Overexpression of UCP1 in tobacco induces mitochondrial biogenesis and amplifies a broad stress response

**DOI:** 10.1186/1471-2229-14-144

**Published:** 2014-05-28

**Authors:** Pedro Barreto, Vagner Katsumi Okura, Izabella Agostinho Pena Neshich, Ivan de Godoy Maia, Paulo Arruda

**Affiliations:** 1 Centro de Biologia Molecular e Engenharia Genética, Universidade Estadual de Campinas (UNICAMP), 13083-875 Campinas, SP, Brazil; 2 Departamento de Genética e Evolução, Instituto de Biologia, Universidade Estadual de Campinas (UNICAMP), 13083-875 Campinas, SP, Brazil; 3 Departamento de Genética, Instituto de Biociências, UNESP, 18618-970 Botucatu, SP, Brazil

**Keywords:** UCP1, Mitochondria, Oxidative stress, Biogenesis, Plant, Stress response

## Abstract

**Background:**

Uncoupling protein one (UCP1) is a mitochondrial inner membrane protein capable of uncoupling the electrochemical gradient from adenosine-5′-triphosphate (ATP) synthesis, dissipating energy as heat. UCP1 plays a central role in nonshivering thermogenesis in the brown adipose tissue (BAT) of hibernating animals and small rodents. A UCP1 ortholog also occurs in plants, and aside from its role in uncoupling respiration from ATP synthesis, thereby wasting energy, it plays a beneficial role in the plant response to several abiotic stresses, possibly by decreasing the production of reactive oxygen species (ROS) and regulating cellular redox homeostasis. However, the molecular mechanisms by which UCP1 is associated with stress tolerance remain unknown.

**Results:**

Here, we report that the overexpression of UCP1 increases mitochondrial biogenesis, increases the uncoupled respiration of isolated mitochondria, and decreases cellular ATP concentration. We observed that the overexpression of UCP1 alters mitochondrial bioenergetics and modulates mitochondrial-nuclear communication, inducing the upregulation of hundreds of nuclear- and mitochondrial-encoded mitochondrial proteins. Electron microscopy analysis showed that these metabolic changes were associated with alterations in mitochondrial number, area and morphology. Surprisingly, UCP1 overexpression also induces the upregulation of hundreds of stress-responsive genes, including some involved in the antioxidant defense system, such as superoxide dismutase (SOD), glutathione peroxidase (GPX) and glutathione-S-transferase (GST). As a consequence of the increased UCP1 activity and increased expression of oxidative stress-responsive genes, the UCP1-overexpressing plants showed reduced ROS accumulation. These beneficial metabolic effects may be responsible for the better performance of UCP1-overexpressing lines in low pH, high salt, high osmolarity, low temperature, and oxidative stress conditions.

**Conclusions:**

Overexpression of UCP1 in the mitochondrial inner membrane induced increased uncoupling respiration, decreased ROS accumulation under abiotic stresses, and diminished cellular ATP content. These events may have triggered the expression of mitochondrial and stress-responsive genes in a coordinated manner. Because these metabolic alterations did not impair plant growth and development, UCP1 overexpression can potentially be used to create crops better adapted to abiotic stress conditions.

## Background

Mitochondrial uncoupling protein one (UCP1) is a nuclear-encoded protein located in the mitochondrial inner membrane. In the presence of fatty acids, UCP1 uncouples the electrochemical gradient from adenosine-5′-triphosphate (ATP) synthesis, dissipating energy as heat [1]. Mammalian UCP1 has long been investigated in brown adipose tissue (BAT) for its role in thermogenesis and the regulation of reactive oxygen species (ROS) production [2-4].

UCP1 has also been found in plants [5]. Similar to its mammalian orthologs, plant UCP1 belongs to a multigenic family whose members are expressed in a time- and tissue-dependent manner and in response to low temperature [6-8]. Plant UCPs have also been shown to be involved in thermogenesis regulation in skunk cabbage [9] and climacteric increases in respiration in fruits [10], but the widespread presence of this protein in eukaryotic organisms suggests that it may have other functions [8]. The overexpression of *Arabidopsis thaliana UCP1* (*AtUCP1*) in tobacco plants resulted in increased tolerance to oxidative stress [11]. In addition, the tobacco plants overexpressing *AtUCP1* exhibited faster germination under control and stressful conditions, improved performance under drought and salt stresses, and increased rates of photosynthesis [12]. The mechanism underlying this increased stress protection is generally associated with decreased ROS production [11,12], but a recent study performed on *Solanum lycopersicum* (tomato) plants overexpressing a UCP gene suggest a wider role for UCPs’ protective mechanisms by altering cell redox homeostasis and antioxidant capacity [13]. These previous studies regarding UCP overexpression, along with a study of an insertional knockout of AtUCP1 [14], suggest that UCPs may alter metabolism more globally, modulating mitochondrial, chloroplastic and cytosolic metabolism.

The role of mitochondria in energy metabolism and the stress response implies that this organelle communicates with other cellular compartments. Alteration of mitochondrial function modulates the expression of UCP1 and other nuclear-encoded mitochondrial proteins, including alternative oxidase (AOx) and type II NAD(P)H dehydrogenase, through mitochondria-to-nucleus signaling [15,16]. Mitochondria-to-nucleus communication has been extensively investigated in mammalian models, in which a signaling process coordinates the expression of genes encoded by the mitochondrial and nuclear genomes through mechanisms known as anterograde (nucleus to organelle) and retrograde (organelle to nucleus) signals [17]. The cytosolic concentrations of Ca^2+^ and ATP, together with the mitochondrial fission/fusion dynamics and ROS production, play central roles in the antero/retrograde signaling pathways and in the regulation of mitochondrial proliferation [18]. Both anterograde and retrograde signaling have been implicated in mitochondrial biogenesis [19,20], a process that is well documented in mammalian models [4] but poorly understood in plants [21]. In plants, the upregulation of nuclear-encoded mitochondrial genes have been seen during inflorescence development, especially during flowering [22-24], but the molecular components involved in the regulation of this process remains elusive. Recently, it has been shown that the pentatricopeptide proteins [25] and the transcription factors bZIP [26], WRKY [27], TCP [28] and NAC [29] may contribute to the retrograde regulation of mitochondrial/nucleus communication in plants.

In the present work, we investigated the mechanism of UCP1 action in the stress response. We used molecular, cellular and genomic tools to investigate the molecular and cellular events resulting from the overexpression of *AtUCP1* in transgenic tobacco plants. We show that *AtUCP1* overexpression increases uncoupled respiration, decreases cellular ATP concentration, alters mitochondrial morphology, and triggers retrograde signaling, activating the expression of mitochondrial- and nuclear-encoded mitochondrial proteins. These changes are accompanied by a broad induction of stress-responsive genes that may help the *AtUCP1*-overexpressing cells reduce the levels of ROS and perform better under stress conditions. The results are discussed in the context of the link between mitochondrial biogenesis and the stress response in plants.

## Results

### Overexpression of the *Arabidopsis UCP1* (*AtUCP1*) in tobacco shows no phenotypic alterations independent of its transgene expression levels

**Figure 1:**
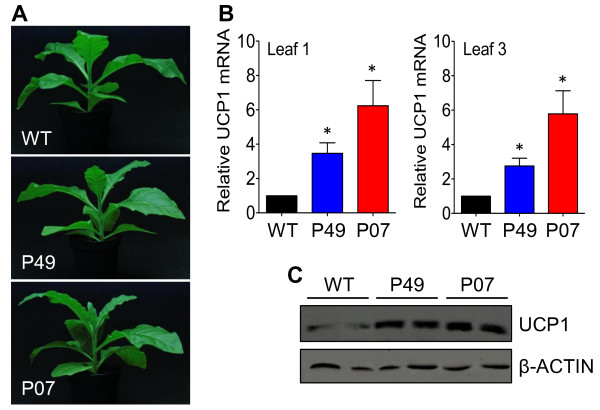
**Transgenic tobacco overexpressing *AtUCP1 *exhibits a normal plant phenotype independent of the UCP1 expression level. (A)** Wild-type (WT) and *AtUCP1* transgenic lines P49 and P07 grown for 12 weeks in 50:50 soil/vermiculite mixtures fertilized with half-strength MS medium not supplemented with sucrose. **(B)** Transcript abundance of *AtUCP1* in L1 and L3 leaves of WT and transgenic lines P49 and P07. **(C)** Immunoblot analysis of L3 leaves of WT and transgenic lines P49 and P07 using an anti-*AtUCP1* antibody. The loading control was examined using an anti-β-actin antibody. Panel **(A)** is representative of an experiment using 12 plants for each genotype (n = 12). Panel **(B)** is representative of an experiment with 6 plants for each genotype (n = 6). Panel **(C)** is representative of an experiment conducted in triplicate. *p < 0.05 compared with the control. Error bars, mean ± s.e.m.

The *AtUCP1* gene, encoding the *Arabidopsis* ortholog of mammalian UCP1 [6], was cloned under the control of the 35S CaMV promoter and transformed into tobacco plants [11]. Two transgenic events, P49 and P07, which presented intermediary and high *AtUCP1* expression, respectively, were chosen for this study. Although UCP1 acts directly in the mitochondrial respiratory chain by uncoupling the electron transport from ATP synthesis, no apparent phenotypic alterations were observed in the P49 and P07 transgenic lines compared with the wild-type (WT) plants (Figure 1A). This result diverges from previous findings showing increased shoot dry mass in plants overexpressing the same UCP1 [12], but it is consistent with observations of tomato plants overexpressing a UCP, which did not exhibit growth stimulation [13]. The addition of sucrose to the growth medium may have altered mitochondrial metabolism and biogenesis [24]. In the data presented in Figure 1A, we did not add sucrose to the nutrient solution.

The transgenic lines P49 and P07 presented 2- to 8-fold increases in *AtUCP1* expression compared to WT plants in the two leaves sampled (Figure 1B). Immunoblotting using an anti-*At*UCP1 polyclonal antibody, which recognizes the tobacco and *Arabidopsis* UCP1, showed that the *At*UCP1 protein (approximately 32 kD) was weakly detectable in WT leaves but accumulated at high levels in P49 and P07 transgenic lines (Figure 1C).

### *AtUCP1* overexpression triggers mitochondria biogenesis through retrograde signaling

**Figure 2:**
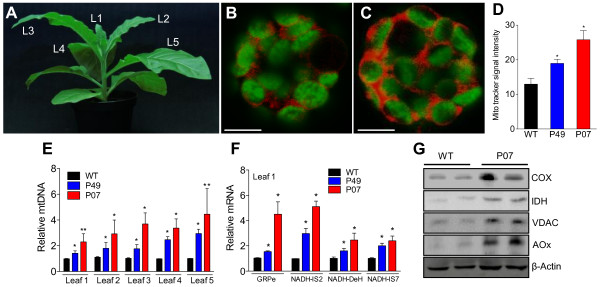
**Overexpression of *AtUCP1 *in tobacco induces mitochondrial biogenesis through retrograde signaling. (A)** Five leaves, from youngest (L1) to oldest (L5), were sampled from WT and transgenic lines P49 and P07. **(B and C)** Mesophyll protoplasts isolated from WT **(B)** and P07 **(C)** L3 leaves stained with MitoTracker Red. The panels are representative of 50 protoplasts evaluated for each genotype. Scale bars = 5 μm. **(D)** Quantification of the MitoTracker Red signal in a population of protoplasts isolated from the L3 leaves of WT and transgenic lines P49 and P07 (n = 50). **(E)** Relative mtDNA content of WT and transgenic lines P49 and P07 in leaves L1 to L5. **(F)** Relative mRNA content of genes involved in targeting proteins to mitochondria (GRPe chaperone) and energy metabolism (NADH-DeH, NADH-IS2, and NADH-IS7) in L1 leaves of WT and transgenic lines P49 and P07. **(G)** Immunoblot analysis of nuclear- (IDH, VDAC and AOX) and mitochondrial-encoded (COXII) proteins involved in energy metabolism, transport, and stress response in the L1 leaves of WT and transgenic line P07. The data presented in panels **E** and **F** are representative of four biological replicates (n = 4). *p < 0.05 and **p < 0.1 compared with WT. Error bars, mean ± s.e.m.

Young and mature leaves differ in their responses to cold stress and photosynthetic activity [30,31]. Therefore, we examined whether young or more expanded leaves would respond differently to *AtUCP1* overexpression. P07, P49 and WT plants were grown for 12 weeks, and apical leaves varying from youngest (L1) to oldest (L5) (Figure 2A) were sampled. Mesophyll protoplasts were prepared from L3, stained with the mitochondria-selective probe MitoTracker Red CM-H2XRos (Invitrogen, Carlsbad, CA, USA) and analyzed with fluorescent confocal microscopy. Both P49 and P07 transgenic lines showed 1.5- to 2-fold increases in the MitoTracker Red fluorescence signal compared with WT, indicating that UCP1 overexpression led to an increase in the mitochondrial number and/or volume (Figure 2B, C, and D). The *Arabidopsis* mitochondrial DNA (mtDNA) encodes 57 proteins involved in mitochondrial metabolism and mtDNA maintenance [32]. In mammals, mtDNA replication is strongly associated with cellular demand for ATP [33]. To investigate whether the increased MitoTracker Red fluorescence signal observed in the P49 and P07 transgenic lines is associated with alteration in mtDNA, we used the *MATR* maturase gene to quantify its mtDNA content relative to the WT line. qRT-PCR quantification of *MATR* in total DNA extracted from L1 to L5 leaves revealed an estimated mtDNA increase of 2- to 5-fold in the P49 and P07 transgenic lines, respectively, compared with that of WT plants (Figure 2E). Because mitochondrial biogenesis requires the coordinated expression of both nuclear and mitochondrial genomes [17,24], we examined whether *AtUCP1* overexpression results in the upregulation of other nuclear- and mitochondrial-encoded mitochondrial proteins. q-PCR of total RNA extracted from L1 to L5 leaves from P49, P07 and WT plants was used to quantify relative mRNA content from genes encoding a selected set of nuclear- and mitochondrial-encoded mitochondrial proteins. Independent of leaf age, the P49 and P07 transgenic lines presented 2- to 5-fold increases in the transcript abundance of NADH dehydrogenase (NADH-DeH) and NADH iron-sulfur proteins 2 and 7 (NADH IS2-7), which are components of mitochondrial respiratory chain complex I [34] (Figure 2F). The increases in both nuclear-encoded (NADH-DeH and NADH-IS7) and mitochondrial-encoded (NADH-IS2) components of complex I indicate that mitochondrial/nuclear coordination is necessary for bioenergetic adaptation. mIncreased transcript abundance in P49 and P07 transgenic lines was also observed for the molecular chaperone GRPe and for the adenine nucleotide exchange factor of heat shock protein-70 (HSP70) that is involved in mitochondrial targeting of proteins [35] (Figure 2F and Additional file 1: Figure S1A-D). We also observed that the P07 transgenic line showed 2- to 5-fold increases in the protein levels of cytochrome oxidase subunit II (COXII), isocitrate dehydrogenase (IDH), voltage-dependent anion channel-1 (VDAC1), and alternative oxidase (AOX), which are proteins associated with both oxidative stress [36] and mitochondrial metabolism [37-39] (Figure 2G). These data indicate that *AtUCP1* overexpression triggered retrograde signaling, leading to increased transcription and translation of mitochondrial proteins encoded by both the nuclear and mitochondrial genomes, culminating in increased mitochondrial biogenesis.

### *AtUCP1* overexpression increases uncoupling respiratory capacity and decreases cellular ATP content

**Figure 3:**
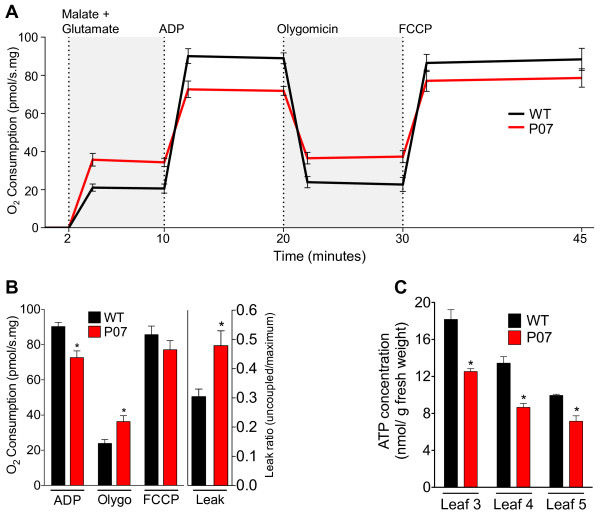
**Overexpression of *AtUCP1 *increases mitochondrial uncoupled respiration, resulting in decreased cellular ATP content. (A)** Time-course of respiration measurements on isolated mitochondria from WT and P07 plants. Malate (10 mM) and glutamate (5 mM) were added to the respiration medium to provide substrates for the electron transport chain. ADP (800 μM) was used to stimulate ADP-dependent respiration, and the ATP synthase inhibitor oligomycin (5 μM) was used to measure oxygen consumption due to uncoupled respiration. Maximum oxygen consumption was measured in the presence of FCCP (2 μM). **(B)** Average oxygen consumption of WT and P07 isolated mitochondria in the presence of ADP, oligomycin and FCCP. The leak ratio was calculated as the ratio between oxygen consumption in the presence of oligomycin and that in the presence of FCCP. **(C)** Decreased ATP levels in the L3, L4 and L5 leaves of the P07 transgenic line compared with WT. The data are representative of six biological replicates (n = 6). *p < 0.05 and **p < 0.1 compared with WT. Error bars, mean ± s.e.m.

Although *AtUCP1* overexpression protects tobacco plants from oxidative, osmotic and drought stress, no previous data indicate whether UCP1 does, in fact, uncouple ATP synthesis in these plants [11]. However, previous work has demonstrated that mitochondria isolated from *Solanum tuberosum* (potato) plants overexpressing a UCP show increased uncoupled respiration [40]. Here, we demonstrate that crude mitochondria isolated from L3 leaves of WT and P07 plants retained their respiratory properties: ADP and FCCP can efficiently increase oxygen consumption when compared to basal respiration (Figure 3A). Additionally, transgenic plants showed decreased adenosine 5′-diphosphate (ADP)-dependent respiration and increased uncoupling respiration in comparison to WT plants (Figure 3B). There was no significant difference in the oxygen consumption in the presence of carbonyl cyanide-*4*-trifluoromethoxy phenylhydrazone (FCCP) between P07 and WT mitochondria (Figure 3B). The increased uncoupling respiration of P07 compared with WT mitochondria is evident in a comparison of the leak ratios (uncoupled/maximum oxygen consumption) in P07 and WT (Figure 3B). The increased uncoupling respiration in the P07 transgenic line resulted in 20–35% decreased cellular ATP concentration in the L3 leaves compared with the WT (Figure 3C).

### Altered mitochondrial metabolism due to increased UCP1 activity leads to changes in mitochondrial number, volume and morphology

**Figure 4:**
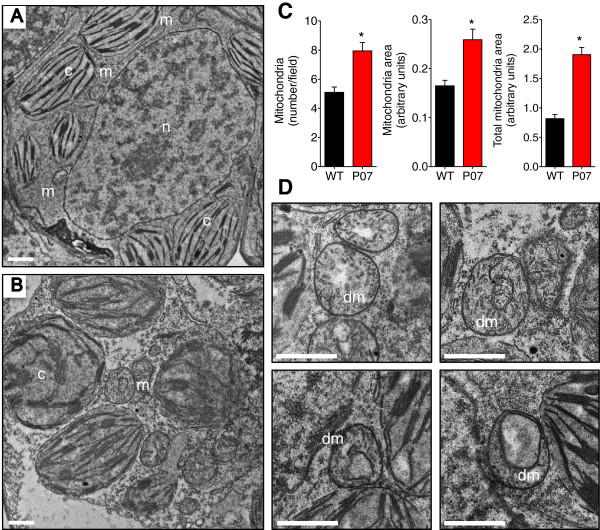
**Overexpression of *AtUCP1 *increased mitochondrial biogenesis through donut formation/fragmentation. (A)** Representative TEM micrograph of WT L2 leaves. **(B)** Representative TEM micrograph of P07 L2 leaves. **(C)** Quantification of the mitochondrial number, area, and total area occupied by mitochondria in TEM micrograph field images of WT and P07 plants. **(D)** Close-up of the donut-shaped mitochondria at different developmental stages in P07 transgenic cells. Image analysis was performed on 20 field images (n = 20) for each genotype. *p < 0.05 compared with WT. Error bars, mean ± s.e.m. Scale bars, 1 μm.

Mitochondrial fission and fusion dynamics, as well as mitochondrial morphology, have been shown to be directly affected by modulations in the energy demand and nutrient supply in human cells [41]. The fine regulation of these parameters is linked to the adaptation of mitochondrial architecture to metabolic demand. To address whether *AtUCP1* overexpression affects mitochondrial morphology, we performed transmission electron microscopy (TEM) of mesophyll cells of L2 leaves from P07 and WT plants. Approximately 20 micrograph fields for each genotype were analyzed for mitochondrial number and area using ImageJ [42]. Images from P07 leaves revealed increases of 1.6-fold in mitochondrial number and 1.4-fold in mitochondrial volume, resulting in an overall augmentation of 2.2-fold in the total mitochondrial area (Figure 4A, B, and C). Surprisingly, and perhaps for the first time, we observed a novel mitochondrial morphology in plant cells: donut-shaped mitochondria (Figure 4D). This morphology has been observed in human cells during treatment with FCCP or subjection to hypoxic stress [43]. These treatments decrease oxygen availability for ATP production. This unusual morphology has also been observed during reoxygenation and FCCP washout, when cells reoxygenate and ATP levels are partially restored. When ATP levels were completely restored, the donut-shaped mitochondria fragmented, generating new mitochondria. These findings suggest that *AtUCP1* overexpression triggers a retrograde signal that promotes mitochondrial biogenesis. The alterations in both mitochondrial architecture and morphology may be due to metabolic stress imposed by the higher activity of the electron transport chain, thus increasing oxygen consumption, together with the reduced ATP synthesis caused by the *At*UCP1 overexpression. The donut-shaped mitochondrial morphology might be associated with the fusion/fission dynamics but may also be important to alleviate the tension imposed by the increased mitochondrial volume [43].

### *AtUCP1* overexpression induces broad transcriptomic activation of mitochondrial and stress-responsive genes

**Figure 5:**
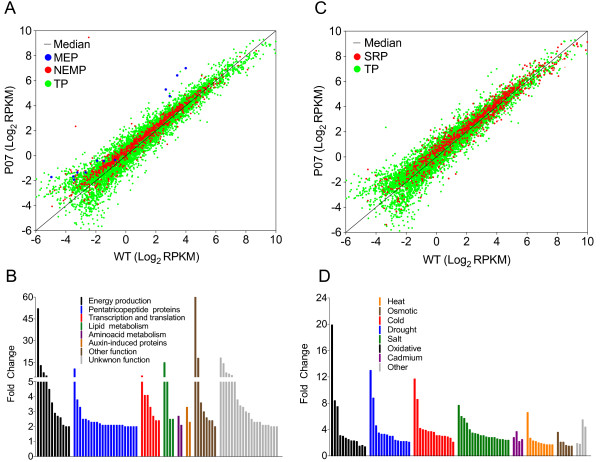
**Transcriptome analysis reveals a global upregulation of mitochondrial and stress-responsive genes in transgenic P07 compared with WT. (A)** Scatterplot of log_2_ RPKM showing nuclear- (Red circle symbol) and mitochondrial (Blue circle symbol)-encoded mitochondrial proteins that were upregulated in the P07 *AtUCP1* transgenic line compared with WT. The green dots represent the total differentially expressed genes. **(B)** Examples of genes associated with nuclear and mitochondrial-encoded mitochondrial proteins that were upregulated in the P07 transgenic line compared with WT. **(C)** Scatterplot of log_2_ RPKM showing stress-responsive (Red circle symbol) genes that were upregulated in *AtUCP1* transgenic lines compared with WT. The green dots represent the total differentially expressed genes. **(D)** Examples of genes associated with abiotic stress responses that were upregulated in the P07 transgenic line compared with WT. Normalization was performed by applying log_2_ to the RPKM values of WT and the P07 transgenic line. All the represented transcripts showed significantly different expression between the genotypes (p < 0.05). The RPKM means were calculated using four biological replicates (n = 4) of L3 leaves from each genotype.

It was unexpected that the overexpression of a single protein would result in mitochondrial biogenesis because the mitochondrial proteome is composed of over 2000 proteins [24]. Therefore, the induction of mitochondrial biogenesis would require the expression of an array of genes encoding these proteins. To examine the extent of retrograde signaling activation due to *AtUCP1* overexpression, we conducted a global transcriptomic analysis of L3 leaves from P07 and WT plants. The transcripts were annotated using UniProt [44] and categorized into the Clustering of Orthologous Groups (COG) database [45]. We used TAIR subcellular prediction of proteins [46] to identify a total of 705 nuclear-encoded mitochondrial transcripts that have significantly higher RPKM values in the P07 transgenic line and 165 that have higher expression levels in WT plants (Figure 5A, red dots). In addition, we identified 12 mitochondrial-encoded proteins, all of which were more than 2-fold (significantly) upregulated in the P07 transgenic line (Figure 5A, blue dots). The most representative mitochondrial upregulated proteins include those associated with energy production, pentatricopeptide proteins, proteins associated with mitochondrial transcription and translation, and a large number of unknown proteins (Figure 5B and Additional file 2: Table S1). Proteins with unknown subcellular prediction were submitted to further analysis using TargetP [47]. This analysis allowed us to identify another 43 proteins predicted to be targeted to mitochondria with fold-changes higher than 2.0 in P07 plants (Additional file 3: Table S2). Among several proteins with unknown function, we identified possible orthologs of one of the mitochondrial calcium uniporters (MCUs) and a mitochondrial sodium/calcium exchanger (MCX). One of these proteins (Solyc04g079910.2.1 in Additional file 3: Table S2), which is 2.5-fold upregulated in P07, contains a coiled-coil domain on the C-terminal region, which is found among the MCUs, and has a strong sequence similarity with the *Arabidopsis thaliana* (*At*MCU), *Mus musculus* (MmMCU) and *Homo sapiens* (HsMCU) mitochondrial calcium uniporters. The other protein (Solyc07g042000.2.1 in Table 2) contains a sodium/calcium-exchanging domain whose sequence is predicted to be targeted to mitochondria. Among the upregulated proteins in the P07 transgenic line, we also identified several proteins associated with calcium signaling, mitochondrial import machinery and mitochondrial fission and fusion processes, along with transcripts related to lipid metabolism, which may act to support UCP1 activity and cell metabolism (Additional file 4: Table S3). Several transcription factors that may play a role in mitochondrial retrograde signaling were also identified (Additional file 4: Table S3). Surprisingly, we also identified 1071 genes responsive to abiotic stress, 72% (770) of which have increased expression in the P07 transgenic line compared with WT (Figure 5C, red dots). Among these stress-associated proteins, we identified large numbers associated with heat, osmotic, cold, drought, salt, oxidative and cadmium stress (Figure 5D). We also observed 1.4- to 3.4-fold increases in the transcript levels of genes involved in antioxidant mechanisms, including SOD, GPX and GSTs, among the upregulated stress-responsive genes (Additional file 5: Table S4).

### UCP1 transgenic line performed better under abiotic stresses

**Figure 6:**
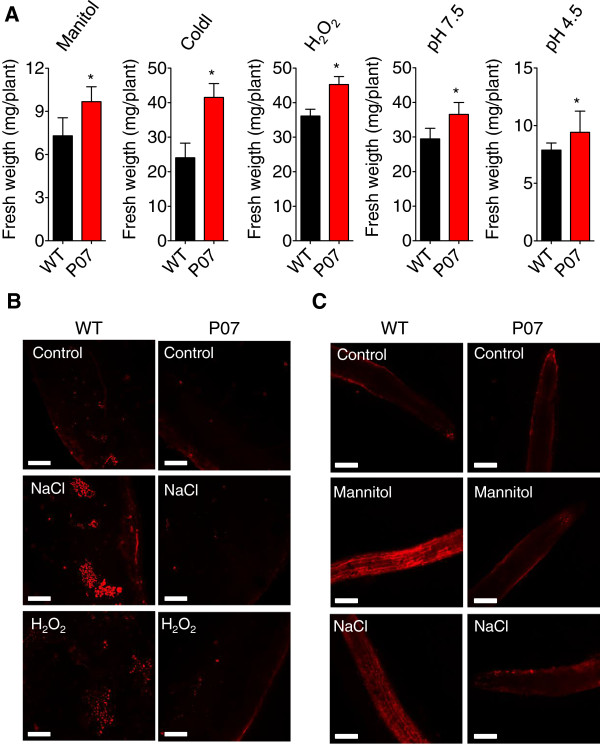
***AtUCP1*-overexpressing plants performed better under abiotic stress, most likely by decreasing ROS accumulation. (A)** Two-week-old WT and P07 plantlets were plated on half-strength MS medium supplemented with 80 mM mannitol (hyperosmotic stress) or 10 mM H_2_O_2_ (oxidative stress), pH 7.5 and pH 4.5 for 22 days under a day/light regime of 16 h at 100 μE m^−2^ s^−1^ and 8 h in darkness. For the low-temperature treatment, the seeds were germinated and grown for 30 days on half-strength MS medium supplemented with 50 μM:10 μM linoleic acid:bovine serum albumin. Plant growth assays were performed using four biological replicates (n = 4) on plates containing 5 plants for each treatment. *p < 0.05 and **p < 0.1 compared with WT. Error bars, mean ± s.e.m. Scale bars, 200 μm. **(B)** Leaf discs (0.8 cm) or **(C)** root tips isolated from WT and the P07 transgenic line were incubated in half-strength MS medium supplemented with 100 mM NaCl, 100mM mannitol, or 10 mM H_2_O_2_ for 20 h. Control treatments were performed using unsupplemented half-strength MS medium. Tissues were stained with MitoSox red and visualized with fluorescent confocal microscopy.

In addition to the previous observation of increased tolerance to oxidative, salt, and drought stresses [11,12], we observed that the P07 UCP1 overexpressor performed better under low and high pH, hyperosmotic stress, and oxidative stress (Figure 6A). Additionally, in the presence of free fatty acids, the P07 transgenic line also exhibited improved performance under low temperature, a result that support the thermogenic role of plant UCP1 (Figure 6A). Because UCP1 activity in mouse brown adipose tissue, potato tuber and tobacco leaves can reduce superoxide accumulation [12,40,48], we examined whether this process also occurs in the P07 transgenic line but used an approach that detects ROS accumulation only within mitochondria. When subjected to salt, oxidative and hyperosmotic stress, mitochondrial ROS accumulation was decreased in the P07 transgenic line compared with WT plants in both leaf discs and roots (Figure 6B and C).

## Discussion

The mitochondrial respiratory chain is composed of four macromolecular complexes through which free energy is conserved by coupling electron transport to the formation of a proton motive force by complexes I, III, and IV, which is then dissipated by F1FO-ATPase (complex V) for ATP synthesis [49]. In the BAT, electron transport is uncoupled from ATP synthesis by UCP1 for heat production [4]. However, BAT is a specialized tissue dedicated to thermogenesis regulation whose main characteristic is a dramatic increase in mitochondria number and UCP1 expression [50]. In other cell types, however, decreased ATP concentration may compromise cell metabolism [51]; therefore, cells somehow sense the cytoplasmic ATP concentration as a signal to change gene expression to quickly readjust the mitochondrial architecture and cell metabolism [18,52]. In this work, we show that the overexpression of UCP1 increases uncoupled respiration and decreases cellular ATP concentration. There is evidence demonstrating that mitochondria isolated from potato plants overexpressing UCP exhibit increased uncoupling respiration [40], but the present study represents the first time that the effect of UCP on intracellular ATP level has been quantified in plants. It is possible that the enhanced uncoupled respiration and its consequent negative effect on the ATP production trigger a signal to compensate for the energy metabolism and maintain ATP at safe levels. Consistent with this view is the remarkable upregulation of electron transport chain components, including NADH-DeH, NADH-IS2-7, and COX, observed in the P07 overexpressor. The coordinated upregulation of the electron transport chain components encoded by the nuclear and mitochondrial genomes occurs in tissues with high metabolic demands and increased mitochondrial biogenesis [21,50,53], such as flowers [22-24,54] and germinating seeds [55].

Mitochondrial biogenesis was observed using multiple approaches, including MitoTracker RED fluorescence imaging, quantitation of mtDNA and TEM. In all cases, these methods allowed us to conclude that the overexpression of *AtUCP1* resulted in increased mitochondrial number and volume. To assess whether the upregulation of mitochondrial genes was restricted to the electron transport chain components, we performed global transcriptome sequencing and observed the upregulation of hundreds of genes encoding mitochondrial proteins. All of the identified transcripts encoded on the mtDNA were upregulated in transgenic plants, which is consistent with the increased mtDNA content. For instance, the ATP synthase gene encoded by the mtDNA was found to be upregulated by 8.1-fold, and two of its nuclear subunits were increased by 2.7- and 3.6-fold. This upregulation was not restricted to the components involved in the oxidative phosphorylation but also included genes that participate in the tricarboxylic acid cycle (TCA). The key control point of the TCA is the enzyme IDH [56], which showed increased protein levels in P07 plants (Figure 2G). In fact, experiments performed with mitochondria isolated from potato plants demonstrated that UCP1 activity facilitates higher TCA flux by decreasing membrane potential and, consequently, ROS production [40].

Transcriptional regulation is the main regulatory factor in the expression of nuclear-encoded genes associated with respiration [21], but the vast majority of proteins targeted to the mitochondria are transported post-translationally by tightly regulated machinery [57]. Among these proteins, HSP70 is involved in the transport from the cytoplasm to the mitochondrial outer membrane, whereas the mitochondrial TOM and TIM translocases are responsible for transport inside the organelle [58]. Interestingly, all of these proteins were upregulated in the P07 *AtUCP1* overexpression line. The increased expression of components of the mitochondrial energy production machinery, along with the increased mitochondrial number of transgenic cells, may alter cellular energy homeostasis. Therefore, the mitochondria may consume more substrates in an attempt to boost ATP synthesis; for that purpose, these substrates must be supplied from other sources. This need might explain the increased photosynthetic rate exhibited by these plants under control conditions [12]. Transcriptome sequencing provides an important tool to investigate this possibility. We did not observe differential expression patterns between WT and P07 plants regarding chloroplast-targeted proteins (data not shown), but further analysis of the transcriptome might provide new insights into this issue.

The adaptation to changes in energy supply and demand influences not only mitochondrial gene expression but also mitochondrial architecture and morphology [41]. Alterations in mitochondrial morphology during starvation have already been observed in *Arabidopsis thaliana* cells cultured without a carbon source [24]. In human cells, treatment with the chemical uncoupler FCCP causes an increase in oxygen consumption and a decrease in ATP generation, provoking mitochondrial fragmentation and the appearance of donut-shaped mitochondria [43]. This fragmentation may occur to support an increase in respiration in an attempt to elevate ATP levels [41], whereas the formation of the donut-shaped morphology may be a component of a protective mechanism that helps to preserve the organelles from damage under conditions of metabolic stress [41,43]. When FCCP is washed out of these cells, the donut morphology persists until the ATP levels are completely restored, at which point the donuts fragment and new mitochondria arise. The mechanisms underlying the donut formation in UCP1-overexpressing cells might be explained by an analogy with human cells. Increased UCP1 activity decreases the membrane potential that is used for ATP synthesis, provoking an increase in mitochondrial number to balance the ATP levels in transgenic plants. Mitochondria are responsible for at least 90% of cell oxygen consumption [59]. We therefore expect that an increase in respiratory chain machinery and mitochondrial number, together with the constant leak caused by UCP1, would result in increased oxygen consumption per molecule of ATP generated. Thus, this metabolic stress caused by UCP1 overexpression appears sufficient to allow the formation of donut mitochondria in plant cells.

Communication between mitochondria and the nucleus is required for the adaptations that we observed in P07 transgenic plants. Although several important regulators of chloroplastic retrograde signaling have been extensively studied [60], relatively little is known about the regulators of mitochondrial retrograde signaling in plants. Several recently published studies suggest roles for the WRKY, bZIP, TCP, and NAC transcription factor families in activating the expression of nuclear-encoded mitochondrial proteins in plants [26-29]. These transcription factors recognize elements that are overrepresented in the regulatory region of mitochondrial genes and that are involved in several biotic and abiotic stress responses [21]. The colocalization of proteins in mitochondria and the nucleus appears to be an important topic that is very little explored in plants. For example, an *Arabidopsis thaliana* pentatricopeptide protein (PNM1) [25] was identified in both mitochondria and nucleus that may act to coordinate the expression between the two genomes. The increased expression of transcription factors that belong to the WRKY and TCP families, along with the upregulation of a large number of pentatricopeptide proteins in transgenic plants, presents the opportunity to further explore new candidates involved in mitochondrial retrograde signaling. The expression of most transcriptional regulators is further linked to cellular signals and environmental cues [21]. The most studied cellular signals involved in mitochondria-nucleus signaling are ATP, calcium and ROS [18]. Although the role of mitochondria in calcium metabolism has been extensively studied in mammals, little is known about calcium signaling by plant mitochondria [61]. Mitochondria in mammals are able to sequester cytoplasmic Ca^+2^, functioning as a transient calcium store for protective mechanisms [18,62]. Additionally, the Ca^+2^ levels inside the organelle are positively correlated with increased ATP production [63], although Ca^+2^ overaccumulation is linked to the induction of apoptosis in both mammals and plants [61]. Consistent with this relation, it is very interesting that we found two genes upregulated in transgenic plants that are possible orthologs, an MCU and an MCX, along with several genes involved in calcium signaling (Additional file 3: Tables S2 and Additional file 4: Table S3). The MCUs and MCXs have been extensively studied in animal cells, and their expression is tightly linked with mitochondrial calcium transport [64,65].

The main focus of previous studies regarding UCP1 overexpression in plants has been ROS production [11-13,40]. Both potato and tobacco transgenic plants presented decreased superoxide production under abiotic stresses [11,12,40]. Additionally, tomato plants overexpressing UCP1 demonstrate not only an alteration on ROS production but also a possible role of UCP1 in regulating the cellular redox homeostasis [13]. The plants analyzed in the present study showed increased expression not only of the antioxidant defense system, including GPX, GSTs and SOD, but also of an array of stress-responsive genes. This effect may help explain the positive impact of *AtUCP1* overexpression on the tolerance to multiple abiotic stresses. Consistent with previous data, we show in this work that *AtUCP1* overexpression protects plants from a number of abiotic stresses, including high pH, low pH and cold. The fact that transgenic tobacco performs better at low temperatures when the medium is supplemented with fatty acids reinforces the thermogenic role of UCP1 in plants. The importance of ROS in cell signaling is well known [66]; thus, the results regarding decreased mitochondrial ROS production under oxidative, osmotic and salt stresses also reinforce the importance of this molecule in UCP1-mediated retrograde signaling.

Together, these data indicate that *AtUCP1*-overexpressing lines suffer metabolic stress caused by the increased uncoupling respiration. Chronic overexpression of UCP1 can induce mitochondrial biogenesis in mammals [67]. Therefore, we propose that in the transgenic plants, the lower ATP level acts as a key element in the retrograde signaling, promoting a broad increase in the expression of both mitochondrial genes and stress-related genes. This pattern is reflected in the decreased ROS production and the better performance of these plants under various stresses. These findings advance our understanding of stress-tolerance mechanisms, mitochondrial biogenesis and bioenergetic adaptation in plants and therefore might assist in the implementation of biotechnological tools for the development of abiotic stress-tolerant plants. The overall regulation of stress-responsive genes indicates a link between UCP1-activated mitochondrial biogenesis and the increased stress response exhibited by the transgenic plants.

## Conclusions

In this work, we present strong evidence that the overexpression of UCP1 protein affects the mitochondrial dynamics at both structural and metabolic levels, leading to an increased mitochondria number and volume per cell. It could be argued that these changes are a consequence of the overpopulation of UCP1 protein at the inner mitochondrial membrane, thereby leading to abnormal mitochondrial structure and function. We interpret the results presented in this manuscript as a beneficial effect of the overexpression of UCP1 that triggers a retrograde signaling process, signaling the nuclear and mitochondrial genomes to increase the production of mitochondrial proteins. Consequently, more mitochondrial membrane is produced. The increase in UCP1, both because of the overproduction of the protein and the increased mitochondrial number and volume, leads to increased uncoupled respiration, decreasing the cellular ATP concentration. This change may trigger metabolic stress and enhances a strong stress response. Because it does not affect plant growth and development, this mechanism might be used to create crops better adapted to abiotic stress conditions.

## Methods

### Plant materials and growth conditions

Tobacco (*Nicotiana tabacum*) ecotype SR1 overexpressing *AtUCP1* was obtained as previously described [11]. In this work, we used two independent transgenic lines, P49 and P07, which express *AtUCP1* at intermediate and high levels, respectively. Transgenic and wild-type (WT) plants were grown under a light/dark cycle of 16 h at 100 μE m^−2^ s^−1^ and 8 h in darkness for 12 weeks on 50:50 soil/vermiculite mixtures at 24°C. The plants were fertilized weekly with half-strength MS medium not supplemented with sucrose. For the cellular and molecular analysis, samples of the five youngest leaves (L1 to L5) of each genotype were collected, frozen in liquid nitrogen, and stored at −80°C. For the other experiments, seeds were grown directly in experimental medium, as described in the figure legends.

### qRT-PCR

For mitochondrial DNA quantification, total DNA was isolated from WT, P49, and P07 plants using the plant DNAzol reagent (Invitrogen, Carlsbad, CA, USA) according to the manufacturer’s protocol. For the mRNA expression analysis, total RNA was isolated from leaf tissue using the NucleoSpin Mini RNA/Protein Kit (Macherey-Nagel, Duren, Germany). cDNA was synthesized using the Revertaid First Strand cDNA Synthesis Kit (Fermentas, Vilnius, Lithuania) according to the manufacturer’s protocol. qRT-PCR was performed using an ABI PRISM 7500 (Applied Biosystems, Foster City, CA, USA) with SYBR Green (Applied Biosystems). The reactions were performed at least in triplicate for four biological replicates. The mRNA expression and mtDNA content analysis were normalized using *ACTIN1* as an internal reference. Values are presented as the mtDNA content or transcript abundance of P49 and P07 relative to WT. Student’s t-test was performed to determine significance (p < 0.05). Primers based on the *MATR* gene were used for mitochondrial DNA quantification [68].

The following primers were designed for amplification:

ACTIN1

FW: 5′-ACTGTCCACGAGGTCCGG-3′

RV: 5′-TGTCGGATCTTGCGCGGC-3′

AtUCP1

FW: 5′-TTGAGCAAGAAAATTCTTGCTG-3′

RV: 5′-AGGCGGAAGGAAAATTAGC-3′

NADH-DeH

FW: 5′-GGCTGAAGCGCGAGAAGAC-3′

RV: 5′-CAGGGCAGGCTTCTTGGC-3′

NADH-IS2

FW: 5′-GTGAAGTGGCGTGGCAAAC-3′

RV: 5′-TTGTGGATCGCGAAGGGAG-3′

NADH-IS7

FW: 5′-CGCAGTTGCGAAGCGAAAC-3′

RV: 5′-GGGCCATCGCGACAGAGA-3′

*GRPe* chaperone

FW: 5′-AAACCTTGGCTTGTGACCCA-3′

RV: 5′-TCATTCGGCCAGCTGAAGTT-3′

### Immunoblot analysis

Total soluble proteins were extracted from WT, P49 and P07 plants by grinding frozen leaf tissue in 5× protein sample buffer (40 mM Tris, pH 7.4; 30 mM NaCl; 10 mM β-mercaptoethanol; 0.1% Triton X-100; 5 mM benzamidine). The proteins were separated on 12% SDS-PAGE gels, transferred to nylon membranes, and blotted using the following polyclonal antibodies raised against *Arabidopsis* proteins (Agrisera, Vännäs, Sweden): COXII, VDAC (At3g01280), AOx1/2 (At2g22370 and At5g64210), IDH (At4g35260 and At2g27130), and ACTIN (At2g37620, At3g18780, At3g53750, At5g59370, At2g42100, At5g09810, At1g49240, At3g12110, and At3G46520). The bands were detected using an ImageQuant LAS500 (GE Healthcare, Little Chalfont, UK) with the SuperSignal West Pico chemiluminescent substrate (Thermo-Scientific, Waltham, MA, USA).

### Mitochondrial quantification in leaf mesophyll protoplasts by MitoTracker Red staining

Tobacco mesophyll protoplasts were isolated from WT, P49, and P07 plants as previously described [69], with minor modifications. Leaf strips (0.1 cm in length) were vacuum-infiltrated and incubated for 3 h in an enzyme solution (20 mM MOPS, pH 5.7; 0.4 M d-mannitol; 20 mM KCl; 10 mM CaCl_2_; 0.1% BSA; 1.5% cellulase; 0.4% macerozyme). After digestion, the suspension was filtered through 75 μm nylon mesh and centrifuged at 100 × g for 2 min. The protoplasts were resuspended in W5 buffer (2 mM MOPS, pH 5.7; 154 mM NaCl; 125 mM CaCl_2_; 5 mM KCl), incubated on ice for 30 min, and subsequently resuspended in MMG solution (4 mM MOPS, pH 5.7; 0.4 M mannitol; 15 mM MgCl_2_). The protoplasts were then stained with 250 nM MitoTracker Red CM-H2XRos (Invitrogen) for 30 min in the dark and washed in MMG solution before viewing. MitoTracker Red CM-H2XRos fluorescence was detected at Ex/Em of 543/589 nm and chlorophyll auto fluorescence was detected at Ex/Em 543/645 nm using a confocal microscope (LSM780-NLO; Zeiss, Oberkochen, Germany) equipped with a 40× oil-immersion objective. The image analysis was performed for 40 protoplasts per sample using ImageJ 1.44p [42]. MitoTracker Red CM-H2XRos fluorescence intensity was normalized using the chlorophyll autofluorescence intensity.

### Mitochondrial superoxide detection by MitoSox staining

Leaf discs (0.8 cm in diameter) isolated from L3 leaves were treated with half-strength MS medium (control) or half-strength MS medium supplemented with 100 mM NaCl (salt stress) or 10 mM H_2_O_2_ (oxidative stress) for 20 h under constant light and agitation. For root analysis, plants grown for 3 weeks on half-strength MS medium were transferred to petri dishes containing fresh half-strength MS medium not supplemented (control) or supplemented with 100 mM NaCl (salt stress) or 100 mM mannitol (hyperosmotic stress) and kept under constant light for 20 h. The leaf discs and roots were stained with 5 μM MitoSox-Red (Invitrogen) for 25 min and washed with half-strength MS medium before viewing. Fluorescence was detected at Ex/Em of 510/580 nm using a confocal microscope (LSM780-NLO; Zeiss) equipped with a 10× objective.

### Transmission electron microscopy

Leaf strips (1–2 mm in length) from L2 leaves of WT and P07 plants were submerged and vacuum infiltrated for at least 2 h in fixative solution (2.5% glutaraldehyde, 2.5% paraformaldehyde in 0.1 M sodium cacodylate buffer, pH 7.4). The samples were washed in 0.1 M cacodylate buffer and postfixed in a solution containing 1% osmium tetroxide (OsO_4_) and 1.5% potassium ferrocyanide (KFeCN_6_) for 1 h. The samples were then washed 3 times in water and incubated in 1% uranyl acetate in maleate buffer for 1 h; this was followed by 3 washes in maleate buffer and subsequent dehydration in a graded ethanol series (50%, 70%, and 90%, 10 min each; 100%, 2 × 10 min). The samples were then placed in propylene oxide for 1 h and infiltrated in a 1:1 mixture of propylene oxide and TAAB 812 Resin mixture (Marivac Canada, St. Laurent, Canada). The samples were embedded in TAAB 812 Resin mixture and polymerized at 60°C for 48 h. Ultrathin sections of approximately 90 nm were cut using a Reichert Ultracut-S microtome, placed onto copper grids, stained with lead citrate, and examined using a Tecnai G2 Spirit BioTWIN transmission electron microscope. The images were recorded with an AMT 2 k CCD camera. The mitochondrial number and area were quantified on 20 field images representing each genotype at 4800× direct magnification. The image analysis was performed using ImageJ 1.44p [42].

### ATP measurement

ATP assays were performed on L4 and L5 leaves isolated from four biological replicates of WT and P07 plants. ATP extraction was performed as previously described [70], with minor modifications. The leaves were ground in liquid nitrogen, and 30 mg of leaf tissue was homogenized with 300 μL of 0.1 M HCl for 5 min. The homogenate was centrifuged at 20,000 × g for 10 min, and the supernatant was centrifuged using a microconcentrator Ultra-15 (Millipore, Billerica-USA) at 14,000 × g for 20 min. The ATP content was determined by using an ATP assay kit (Invitrogen) and a GloMax luminometer (Promega, Fitchburg, WI, USA).

### Measurement of mitochondrial respiration

Measurements of mitochondrial respiration were performed using crude isolated mitochondria from six biological replicates of WT and P07 plants. The isolation of crude mitochondria was performed as previously described [71], with minor modifications. All steps were performed at 4°C. Approximately 5 g of leaves were cut with a razor blade and ground in 20 mL of grinding buffer (0.3 M sucrose, 60 mM TES, 10 mM EDTA, 10 mM KH_2_PO_4_, 25 mM disodium pyrophosphate, 1 mM glycine, 1% (w/v) PVP-40, 1% (w/v) BSA, 50 mM sodium ascorbate, 20 mM cysteine, pH 8.0). The extract was filtered through two layers of 20 μm nylon mesh and one layer of Miracloth (Millipore, Billerica, MA, USA) and centrifuged twice at 2500 × g for 5 min to remove the debris. The resulting supernatant was centrifuged at 15,000 × g for 15 min, and the resulting pellet was gently resuspended in 20 mL of wash buffer (0.3 M sucrose, 10 mM TES, 2 mM EDTA, 10 mM KH_2_PO_4_, pH 7.5) and centrifuged at 15,000 × g for 15 min. The pellet containing the crude mitochondria was resuspended in 200 μL of wash buffer. The respiratory measurements were performed using an Oxygraph-2 k respirometer (Oroboros, Innsbruck, Austria) at 25°C in 2 mL of mitochondria-containing assay buffer (0.3 M sucrose, 10 mM TES, 10 mM KCl, 2 mM MgSO_4_, 5 mM KH_2_PO_4_, 0.1% (w/v) BSA, 2 μM EGTA, pH 7.5). Malate (5 mM) and glutamate (10 mM) were used as substrates to stimulate respiration. ADP (800 μM) was added to measure ADP-dependent respiration. Oligomycin (5 μM) was used as an F1FO ATP synthase inhibitor to measure oxygen consumption due to uncoupling activity, and the chemical ionophore carbonyl cyanide-*4*-(trifluoromethoxy)phenylhydrazone (FCCP) (2 μM) was used to determine the maximum oxygen consumption.

### Transcriptome sequencing, assembly, and mapping

Total RNA isolated from four biological replicates of WT and P07 L3 leaves was used to create single-end RNASeq libraries using the Illumina TruSeq RNA Sample Prep Kit (Illumina, San Diego, CA, USA) according to the manufacturer’s instructions. The libraries were sequenced in four lanes of an Illumina HiSeq 2000 for 75 cycles. A total of 42.7 Gb of sequence was generated for the 8 libraries, with a minimum of 27.9 million and a maximum of 113.2 million reads for each library (Additional file 6: Table S5). The Illumina reads were filtered to remove adapters and low-quality reads (reads <70% bases with quality ≥ Q20) using AdapterRemoval, the FASTX-Toolkit (http://hannonlab.cshl.edu/fastx_toolkit), and Perl scripts. After the filtering step, the reads were subjected to digital normalization using the diginorm software (https://github.com/ged-lab/2012-paper-diginorm). The resulting 49.5 million reads were assembled with the Trinity [72] assembler, generating 271,750 transcript contigs. High-quality reads (569 million reads) were mapped to Trinity contigs using Bowtie [73], and the RPKM [74] values were calculated. Because the *N. tabacum* genome is not yet completely sequenced and the scaffolds of *N. benthamiana* are estimated to cover only 79% of its genome, we used the complete genome sequence of *Solanum lycopersicum*[75], a close relative of *N. tabacum*, as a template. To identify protein-coding genes, all 271,750 contigs were used as queries in BLASTn and BLASTx searches against both the non-redundant set of 34,727 tomato-coding sequences (CDSs) and predicted protein sequences. The 271,750 contigs were compared to the Solgenomics tomato genome predicted protein database using BLASTx (E-value <1e^−5^). Of the total contigs, 134,752 mapped to the tomato genome, resulting in the identification of 20,045 distinct orthologs for the tomato CDS models. As representative contigs, we chose the three best matching contigs with higher average RPKM values (P07 and WT) for each tomato CDS. An F-test was used to calculate whether the samples had equal or unequal variances, and the appropriate Student t-test was then applied to calculate the significant difference between the average RPKM of P07 versus WT in a contig-wise manner. The contigs identified as differentially expressed were selected for the next steps. The fold change value for the representative transcript was calculated as the ratio between the average RPKM values in P07 and WT. The subcellular annotation of the defined contigs was determined by BLASTx queries against the TAIR [46] protein database (release 10) and TargetP [47]. Gene function data were obtained from the existing annotations of the tomato genome. The tomato protein dataset was annotated against UniProt [44] and mapped against the COG (Eukaryotic Clusters of Orthologous Groups) [45] database with BLASTp, and COG functional categories were assigned only if the two best hits had the same COG function. Stress-related genes were mapped using BLASTx against the tomato proteome in the Stress Responsive Transcription Factor Database (STIFDB) [76], and annotations were assigned to the corresponding representative tobacco contig.

### Accession numbers

Sequence data from this article can be found in the GenBank/EMBL database under the following accession numbers: *At*UCP1 (NM_115271.4), Actin1 (EU938079.1), NADH-DeH (Y09109.1), NADH-IS2 (M77225.1), NADH-IS7 (L16810.1), GRPe chaperone (AF098636.1), *At*MCU (NP_119075), *Mm*MCU (XP_006513531), *Hs*MCU (NP_612366), *At*CCX3 (NP_566474).

Transcriptome sequencing data are available from Sequence Read Archive (SRA), which is accessible through NCBI BioProject ID: PRJNA211804 under the experiment ID SUB287723.

## Competing interests

The authors declare that they have no competing interests.

## Authors’ contributions

P.B. and P.A. designed the experiments, analyzed the data, and wrote the paper. P.B. performed the experiments. V.O. and I.A.P.N. performed the bioinformatics analysis of the RNASeq data. I.G.M. developed the transgenic plants. All authors read and approved the final manuscript.

## Supplementary Material

Additional file 1: Figure S1 Tobacco plants overexpressing *AtUCP1* show an increased expression of nuclear-encoded mitochondrial genes. Click here for file

Additional file 2: Table S1 Classification of mitochondrial genes upregulated in P07 compared to WT. The classification was based on a TAIR annotation of protein subcellular prediction. Click here for file

Additional file 3: Table S2 Predicted mitochondrial genes upregulated with ≥ 2-fold change in P07 compared with WT. The prediction was based on TargetP. Click here for file

Additional file 4: Table S3 Classification of selected genes involved in different metabolic processes and significantly upregulated in P07 compared with WT. The classification was based on COG. Click here for file

Additional file 5: Table S4 Abiotic stress-responsive genes upregulated in P07 compared with WT. The classification was based on STIFDB and shows the most upregulated genes involved in different types of abiotic stresses. Click here for file

Additional file 6: Table S5 Summary of RNASeq data filtered and mapped with Bowtie. Click here for file

## References

[B1] Nedergaard J Ricquier D Kozak LP Uncoupling proteins: current status and therapeutic prospects EMBO Rep 2005 6 917 921 10.1038/sj.embor.7400532 16179945 PMC1369193

[B2] Krauss S Zhang CY Lowell BB The mitochondrial uncoupling-protein homologues Nat Rev Mol Cell Biol 2005 6 248 261 10.1038/nrm1592 15738989

[B3] Andrews ZB Diano S Horvath TL Mitochondrial uncoupling proteins in the CNS: in support of function and survival Nat Rev Neurosci 2005 6 829 840 10.1038/nrn1767 16224498

[B4] Kajimura S Seale P Spiegelman BM Transcriptional control of brown fat development Cell Metab 2010 11 257 262 10.1016/j.cmet.2010.03.005 20374957 PMC2857670

[B5] Vercesi AE Martins LS Silva MAP Leite HMF Cuccovia IM Chaimovich H PUMPing plants Nature 1995 375 24 10.1038/375024a0

[B6] Maia IG Benedetti CE Leite A Turcinelli SR Vercesi AE Arruda P AtPUMP: an Arabidopsis gene encoding a plant uncoupling mitochondrial protein FEBS Lett 1998 429 403 406 10.1016/S0014-5793(98)00634-6 9662458

[B7] Borecky J Nogueira FTS Oliveira KAP Maia IG Vercesi AE Arruda P The plant energy-dissipating mitochondrial systems: depicting the genomic structure and the expression profiles of the gene families of uncoupling protein and alternative oxidase in monocots and dicots J Exp Bot 2006 57 849 864 10.1093/jxb/erj070 16473895

[B8] Vercesi AE Borecký J Maia IG Arruda P Cuccovia IM Chaimovich H Plant uncoupling mitochondrial proteins Annu Rev Plant Biol 2006 57 383 404 10.1146/annurev.arplant.57.032905.105335 16669767

[B9] Ito-Inaba Y Hida Y Mora H Inaba T Molecular identity of uncoupling proteins in thermogenic skunk cabbage Plant Cell Physiol 2008 49 1911 1916 10.1093/pcp/pcn161 18974196

[B10] Jezek P Borecký J Zácková M Costa AD Arruda P Possible basic and specific functions of plant mitochondrial uncoupling protein (pUCP) Biosci Rep 2001 21 237 245 10.1023/A:1013660611154 11725872

[B11] Brandalise M Maia IG Borecký J Vercesi AE Arruda P Overexpression of plant mitochondrial uncoupling protein in transgenic tobacco increases tolerance to oxidative stress J Bioenerg Biomembr 2003 35 205 209 10.1023/a:1024603530043 13678271

[B12] Begcy K Mariano ED Mattiello L Nunes AV Mazzafera P Maia IG Menossi M An *Arabidopsis* mitochondrial uncoupling protein confers tolerance to draught and salt stress in transgenic tobacco plants Plos One 2011 6 e23776 10.1371/journal.pone.0023776 21912606 PMC3166057

[B13] Chen S Liu A Zhang S Li C Chang R Liu D Ahammed GJ Lin X Overexpression of mitochondrial uncoupling protein conferred resistance to heat stress an *Botrytis cinerea* infection in tomato Plant Physiol Biochem 2013 73 245 253 24161754 10.1016/j.plaphy.2013.10.002

[B14] Sweetlove LJ Lytovchenko A Morgan M Nunes-Nesi A Taylor NL Baxter CJ Eickmeier A Fernie AR Mitochondrial uncoupling protein is required for efficient photosynthesis Proc Natl Acad Sci U S A 2006 103 19587 19592 10.1073/pnas.0607751103 17148605 PMC1748269

[B15] Rhoads DM Umbach AL Subbaiah CC Siedow JN Mitochondrial reactive oxygen species: contribution to oxidative stress and interorganellar signaling Plant Physiol 2006 141 357 366 10.1104/pp.106.079129 16760488 PMC1475474

[B16] Rhoads DM Subbaiah CC Mitochondrial retrograde regulation in plants Mitochondrion 2007 7 177 194 10.1016/j.mito.2007.01.002 17320492

[B17] Woodson JD Chory J Coordination of gene expression between organellar and nuclear genomes Nat Rev Genet 2008 9 383 395 10.1038/nrg2348 18368053 PMC4854206

[B18] Ryan MT Hoogenrad MJ Mitochondrial-nuclear communications Annu Rev Biochem 2007 76 701 722 10.1146/annurev.biochem.76.052305.091720 17227225

[B19] Pesaresi P Schneider A Kleine T Leister D Interorganellar communication Curr Opin Plant Biol 2007 10 600 606 10.1016/j.pbi.2007.07.007 17719262

[B20] Jung HS Chory J Signaling between chloroplasts and the nucleus: can a systems biology approach bring clarity to a complex and highly regulated pathway Plant Physiol 2009 152 453 459 19933385 10.1104/pp.109.149070 PMC2815895

[B21] Welchen E Garcia L Mansilla N Gonzalez DH Coordination of plant mitochondrial biogenesis: keeping pace with cellular requirements Front Plant Sci 2013 4 551 24409193 10.3389/fpls.2013.00551 PMC3884152

[B22] Zabaleta E Heiser V Grohmann L Brennicke A Promoters of nuclear-encoded respiratory chain complex I genes from Arabidopsis thaliana contain a region essential for anther/pollen-specific expression Plant J 1998 156 49 59 9744094 10.1046/j.1365-313x.1998.00177.x

[B23] Binder S Brennicke A Gene expression in plant mitochondria: transcriptional and post-transcriptional control Philos Trans R Soc Lond B Biol Sci 2003 3586 181 188 12594926 10.1098/rstb.2002.1179 PMC1693100

[B24] Giegè P Sweetlove LJ Congat V Leaver CJ Coordination of nuclear and mitochondrial genome expression during mitochondrial biogenesis in Arabidopsis Plant Cell 2005 17 1497 1512 10.1105/tpc.104.030254 15829605 PMC1091770

[B25] Hammani K Gobert A Hleibieh K Choulier L Small L Giegè P An *Arabidopsis* dual-localized pentatricopeptide repeat protein interacts with nuclear proteins involved in gene expression regulation Plant Cell 2011 23 730 740 10.1105/tpc.110.081638 21297037 PMC3077779

[B26] Roschzttardtz H Fuentes I Vásquez M Corvalán C León G Gómez I Araya A Holuique L Vicente-Carbajosa J Jordana X A nuclear gene encoding the iron-sulfur subunit of mitochondrial complex II is regulated by B3 domain transcription factors during seed development in *Arabidopsis thaliana* Plant Physiol 2009 150 84 95 10.1104/pp.109.136531 19261733 PMC2675723

[B27] Van Aken O Zhang B Law S Narsai R Whelan J AtWKRY40 and AtWRKY63 modulate the expression of stress-responsive nuclear genes encoding mitochondrial and chloroplast proteins Plant Physiol 2013 162 254 271 10.1104/pp.113.215996 23509177 PMC3641207

[B28] Giraud E Ng S Carrie C Duncan O Low J Lee CP Van Aken O Millar AH Murcha M Whelan J TCP transcription factors link the regulation of genes encoding mitochondrial proteins with the circadian clock in *Arabidopsis thaliana* Plant Cell 2010 22 3921 3934 10.1105/tpc.110.074518 21183706 PMC3027163

[B29] De Clercq I Vermeirssen V Van Aken O Vandepoele K Murcha MW Law SR Inzé A Ng S Ivanova A Rombaut D Van de Cotte B Jaspers P Van de Peer Y Kangasjärvi J Whelan J Van Breusegem F The membrane-bound NAC transcription factor ANAC013 functions in mitochondrial retrograde regulation of the oxidative stress response in Arabidopsis Plant Cell 2013 25 3472 3490 10.1105/tpc.113.117168 24045019 PMC3809544

[B30] Ölçer H Lloyd JC Raines CA Photosynthetic capacity is differentially affected by reductions in sedoheptulose-1,7-biphosphatase activity during leaf development in transgenic tobacco plants Plant Physiol 2001 125 982 989 10.1104/pp.125.2.982 11161054 PMC64898

[B31] Rogalski M Schöttler MA Thiele W Schulze WX Bock R Rpl33, a nonessential plastid-encoded ribosomal protein in tobacco, is required under cold stress conditions Plant Cell 2008 20 2221 2237 10.1105/tpc.108.060392 18757552 PMC2553612

[B32] Unseld M Marienfeld JR Brandt P Brennicke A The mitochondrial genome of *Arabidopsis thaliana* contains 57 genes in 366,924 nucleotide Nat Genet 1997 15 57 61 10.1038/ng0197-57 8988169

[B33] Dickinson A Yeung KY Donoghue J Baker MJ Kelly RDW McKenzie M Jhons TJ St John JC The regulation of mitochondrial DNA copy number in glioblastoma cells Cell Death Differ 2013 20 1644 1653 10.1038/cdd.2013.115 23995230 PMC3824586

[B34] Klodmann J Senkler M Rode C Braun HP Defining the protein complex proteome of plant mitochondria Plant Physiol 2011 157 587 598 10.1104/pp.111.182352 21841088 PMC3192552

[B35] Padimam M Reddy VS Beachy RN Fauquet CM Molecular characterization of a plant mitochondrial chaperone GrpE Plant Mol Biol 1999 39 871 881 10.1023/A:1006143305907 10344193

[B36] Fiorani F Umbach AL Siedow JN The alternative oxidase of plant mitochondria is involved in the acclimation of shoot growth at low temperature. A study of Arabidopsis AOX1a transgenic plants Plant Physiol 2005 139 1795 1805 10.1104/pp.105.070789 16299170 PMC1310560

[B37] Smolková K Jezek P The role of mitochondrial NADPH-dependent isocitrate dehydrogenase in cancer cells Int J Cell Biol 2012 212 1 12 10.1155/2012/273947 PMC3363418 22675360

[B38] Robert N D’Erfurth I Marmagne A Erhardt M Allot M Boivin K Gissot L Monachello D Michaud M Duchêne AM Barbier-Brygoo H Maréchal-Drouard L Ephritikhine G Filleur S Voltage-dependent-anion-channels (VDACs) in Arabidopsis have a dual localization in the cell but show a distinct role in mitochondria Plant Mol Biol 2012 78 431 446 10.1007/s11103-012-9874-5 22294207

[B39] Leadsham JE Sanders G Giannaki S Bastow EL Hutton R Naeimi WR Breitenbach M Gourlay CW Loss of cytochrome c oxidase promotes RAS-dependent ROS production from the ER resident NADPH oxidase, Yno1p, in yeast Cell Metab 2013 18 279 286 10.1016/j.cmet.2013.07.005 23931758

[B40] Smith AMO Ratcliffe G Sweetlove LJ Activation and function of mitochondrial uncoupling protein in plants J Biol Chem 2004 279 51944 51952 10.1074/jbc.M408920200 15456782

[B41] Liesa M Shirihai OS Mitochondrial dynamics in the regulation of nutrient utilization and energy expenditure Cell Metab 2013 17 491 506 10.1016/j.cmet.2013.03.002 23562075 PMC5967396

[B42] Schneider CA Rasband WS Eliceir KW NIH image to ImageJ: 25 years of image analysis Nat Methods 2012 9 671 675 10.1038/nmeth.2089 22930834 PMC5554542

[B43] Liu X Hajnóczky G Altered fusion dynamics underlie unique morphological changes in mitochondria during hypoxia-reoxygenation stress Cell Death Differ 2011 18 1561 1572 10.1038/cdd.2011.13 21372848 PMC3172112

[B44] The UniProt Consortium Update on activities at the universal protein resource (UniProt) in 2013 Nucleic Acids Res 2013 41 D43 D47 23161681 10.1093/nar/gks1068 PMC3531094

[B45] Tatusov RL Fedorova ND Jackson JD Jacobs AR Kiryutin B Koonin EV Krylov DM Mazumder R Mekhedov SL Nikoslkaya AN Rao BS Smirnov AS Sverdlove AV Vasudevan S Wolf YI Yin JJ Natale DA The COG database: an updated version includes eukaryotes BMC Bioinforma 2003 4 41 10.1186/1471-2105-4-41 PMC222959 12969510

[B46] Lamesch P Berardini TZ Li D Swarbreck D Wilks C Sasidharan R Muller R Dreher K Alexander DL Garcia-Hernandez M Karthikeyan AS Lee CH Nelson WD Ploetz L Singh S Wensel A Huala E The Arabidopsis information resource (TAIR): improved gene annotation and new tools Nucleic Acids Res 2012 40 1202 1210 10.1093/nar/gkr1090 PMC3245047 22140109

[B47] Emanuelsson O Brunak S Heijne VJ Nielsen H Locating proteins in the cell using TargetP, SignalP, and related tools Nat Protoc 2007 2 953 971 10.1038/nprot.2007.131 17446895

[B48] Oelkrug R Kutschke M Meyer CW Heldmaier G Jastroch M Uncoupling protein 1 decreases superoxide production in brown adipose tissue mitochondria J Biol Chem 2010 285 21961 21968 10.1074/jbc.M110.122861 20466728 PMC2903373

[B49] Vafai SB Mootha VK Mitochondrial disorders as windows into an ancient organelle Nature 2012 491 374 383 10.1038/nature11707 23151580

[B50] Wu Z Puigserver P Andersson U Zhang C Adelmant G Mootha V Troy A Cinti S Lowell B Scarpulla RC Spiegelman BM Mechanisms controlling mitochondrial biogenesis and respiration through the thermogenic coactivator PGC-1 Cell 1999 98 115 124 10.1016/S0092-8674(00)80611-X 10412986

[B51] Miyoshi N Oubrahim H Chock PB Stadtman ER Age-dependent cell death and the role of ATP in hydrogen-peroxide-induced apoptosis and necrosis Proc Natl Acad Sci U S A 2006 103 1727 1731 10.1073/pnas.0510346103 16443681 PMC1413652

[B52] Moghadam AA Ebrahimie E Taghavi SM Niazi A Babgohari MZ Deihimi T Djavaheri M Ramezani A How the nucleus and mitochondria communicate in energy production during stress: nuclear MtATP6, an early-stress responsive gene, regulates the mitochondrial F₁F₀-ATP synthase complex Mol Biotechnol 2013 54 756 769 10.1007/s12033-012-9624-6 23208548

[B53] Lin J Wu H Tarr PT Zhang CY Wu Z Boss O Michael LF Puigserver P Isotani E Olson EN Lowell BB Bassel-Duby R Spiegelman BM Transcriptional co-activator PGC-1 alpha drives the formation of slow twitch muscle fibres Nature 2002 418 797 801 10.1038/nature00904 12181572

[B54] Peters K Niessen M Peterhansel C Spath B Holzle A Binder S Marchfelder A Braun HP Complex I-complex II ratio strongly differs in various organs of *Arabidopsis thaliana* Plant Mol Biol 2012 79 273 284 10.1007/s11103-012-9911-4 22527752

[B55] Law SR Narsai R Taylor NL Dellanoy E Carrie C Giraud E Millar AH Small I Whelan J Nucleotide and RNA metabolism prime translational initiation in the earliest events of mitochondrial biogenesis during Arabidopsis germination Plant Physiol 2012 158 1610 1627 10.1104/pp.111.192351 22345507 PMC3320173

[B56] Berg JM Tymoczko JL Stryer L Biochemistry The Citric Acid Cycle 2002 5 New York: W H Freeman

[B57] Pfanner N Geissler A Versatility of the mitochondrial protein import machinery Nat Rev Mol Cell Biol 2001 2 339 349 10.1038/35073006 11331908

[B58] Schleiffe E Becker T Common ground for protein translocation: access control for mitochondria and chloroplasts Nat Rev Mol Cell Biol 2011 12 48 59 10.1038/nrm3027 21139638

[B59] Searcy DG Metabolic integration during the evolutionary origin of mitochondria Cell Res 2003 13 229 238 10.1038/sj.cr.7290168 12974613

[B60] Kleine T Leister D Retrograde signals galore Front Plant Sci 2013 4 1 3 23487593 10.3389/fpls.2013.00045 PMC3594843

[B61] Stael S Wurzinger B Mair A Mehlmer N Vothknecht UC Teige M Plant organellar calcium signaling: an emerging field J Exp Bot 2012 63 1525 1542 10.1093/jxb/err394 22200666 PMC3966264

[B62] Clapham DE Calcium signaling Cell 2007 131 1047 1058 10.1016/j.cell.2007.11.028 18083096

[B63] Tarasov AI Griffiths EJ Rutter GA Regulation of ATP production by mitochondrial Ca + 2 Cell Calcium 2012 52 28 35 10.1016/j.ceca.2012.03.003 22502861 PMC3396849

[B64] Alam MR Groschner LN Parichatikanond W Kuo L Bondarenko AI Rost R Waldeck-Weiermaier M Mali R Graier WF Mitochondrial Ca2+ uptake 1 (MICU1) and mitochondrial Ca + 2 uniporter (MCU) contribute to metabolism secretion coupling in clonal pancreatic β-cells J Biol Chem 2012 287 34445 34454 10.1074/jbc.M112.392084 22904319 PMC3464549

[B65] Palty R Silverman WF Herschfinkel M Caporale T Sensi SL Parnis J Nolte C Fishman D Shoshan-Barmatz D Herrmann S Khananshvili D Sekler I NCLX is an essential component of mitochondrial Na^+^/Ca^+2^ exchange Proc Natl Acad Sci U S A 2010 107 436 441 10.1073/pnas.0908099107 20018762 PMC2806722

[B66] Mailloux RJ Harper ME Mitochondrial proticity and ROS signaling: lessons from the uncoupling proteins Trends Endocrinol Metab 2012 23 451 458 10.1016/j.tem.2012.04.004 22591987

[B67] Si Y Palani S Jayaraman A Lee K Effects of forced uncoupling protein1 expression in 3T3-L1 cells on mitochondrial function and lipid metabolism J Lipid Res 2007 48 826 836 10.1194/jlr.M600343-JLR200 17202129

[B68] Wang DY Zhang Q Liu Y Lin ZF Zhang SX Sun MX Sodmergen The levels of male gametic mitochondrial DNA are highly regulated in angiosperms with regard to mitochondrial inheritance Plant Cell 2010 22 2402 2416 10.1105/tpc.109.071902 20605854 PMC2929101

[B69] Yoo SD Cho YH Sheen J Arabidopsis mesophyll protoplasts: a versatile cell system for transient gene expression analysis Nat Protoc 2007 2 1565 1572 10.1038/nprot.2007.199 17585298

[B70] Ogawa K Hatano-Iwasaki A Yanagida M Iwabuchi M Level of glutathione is regulated by ATP-dependent ligation of glutamate and cysteine through photosynthesis in Arabidopsis thaliana: mechanism of strong interaction of light intensity with flowering Plant Cell Physiol 2004 45 1 8 10.1093/pcp/pch008 14749480

[B71] Keech O Dizengremel P Gardestrom P Preparation of leaf mitochondria from *Arabidopsis thaliana* Physiol Plant 2005 124 403 409 10.1111/j.1399-3054.2005.00521.x

[B72] Grabherr MG Haas BJ Yassour M Levin JZ Thompson DA Amit I Adiconis X Fan L Raychowdhury R Zeng Q Chen Z Mauceli E Hacohen N Gnirke A Rhind N di Palma F Birren BW Nusbaum C Lindblad-Toh K Friedman N Regev A Full-length transcriptome assembly from RNA-seq data without reference genome Nat Biotechnol 2011 29 644 652 10.1038/nbt.1883 21572440 PMC3571712

[B73] Wagner GP Kin K Lynch VJ Measurement of mRNA abundance using RNA-seq data: RPKM measure is inconsistent among samples Theory Biosci 2012 131 281 285 10.1007/s12064-012-0162-3 22872506

[B74] Langmead B Trapnell C Pop M Salzberg SL Ultrafast and memory-efficient alignment of short DNA sequences to the human genome Genome Biol 2009 10 R25 10.1186/gb-2009-10-3-r25 19261174 PMC2690996

[B75] Tomato Genome Consortium The tomato genome sequence provides insights into fleshy fruit evolution Nature 2012 485 635 641 10.1038/nature11119 22660326 PMC3378239

[B76] Naika M Shameer K Mathew OK Gowda R Sowdhamini R STIFDB2: An updated version of plant stress-responsive transcription factor database with additional stress signals, stress-responsive transcription factor binding sites and stress-responsive genes in Arabidopsis and rice Plant Cell Physiol 2013 54 1 15 10.1093/pcp/pcs172 23314754 PMC3583027

