# Supplementary material for: Overexpression of UCP1 in tobacco induces mitochondrial biogenesis and amplifies a broad stress response
Source: BMC Plant Biol. 2014 May 28;14:144. doi: 10.1186/1471-2229-14-144 (PMC4046140; doi:10.1186/1471-2229-14-144)
Supplement: Additional file 1: Figure S1 — Tobacco plants overexpressing AtUCP1 show an increased expression of nuclear-encoded mitochondrial genes. [file 1471-2229-14-144-S1.docx]

**Supplemental Figure 1 -** Tobacco plants overexpressing *AtUCP1* show an increased expression of nuclear-encoded mitochondrial genes. (**A**) Leaf 2, (**B**) Leaf 3, (**C**) Leaf 4 and (**D**) Leaf 5. *, p < 0.05, **, p < 0.1, compared with wild type. Error bars, mean ± s.e.m.

**B**

**A**


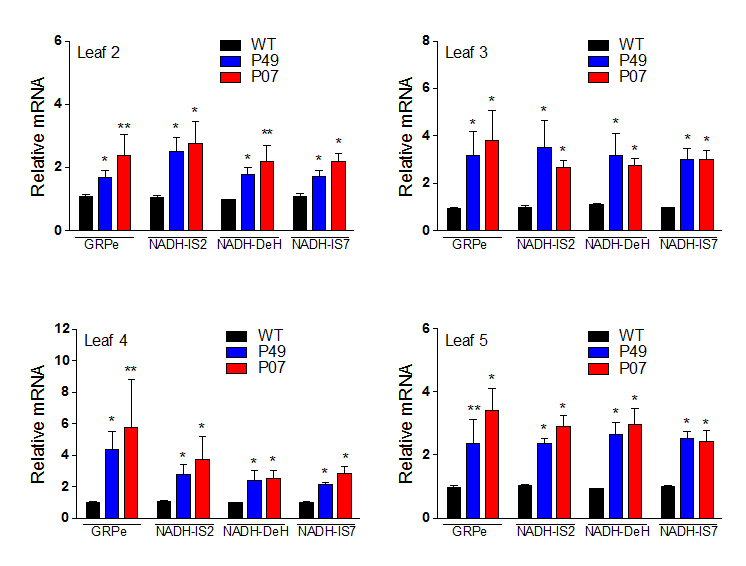


**D**

**C**

**d**
