# Supplementary material for: Overexpression of UCP1 in tobacco induces mitochondrial biogenesis and amplifies a broad stress response
Source: BMC Plant Biol. 2014 May 28;14:144. doi: 10.1186/1471-2229-14-144 (PMC4046140; doi:10.1186/1471-2229-14-144)
Supplement: Additional file 2: Table S1 — Classification of mitochondrial genes upregulated in P07 compared to WT. The classification was based on a TAIR annotation of protein subcellular prediction. [file 1471-2229-14-144-S2.docx]

**Supplemental Table 1.** Classification of mitochondrial genes upregulated in P07 compared to WT. The classification was based on a TAIR annotation of protein subcellular prediction.

| **Classification/Uniprot Description** | ***S. lycopersicum* ID** | **Fold change** | **Encoded by** |
| --- | --- | --- | --- |
| **Energy Production** |  |  |  |
| Mitochondrial glycoprotein family protein | Solyc06g065800.2.1 | 52,1 | Nucleus |
| Mitochondrial 2-oxoglutarate/malate carrier | Solyc05g051400.2.1 | 13,0 | Nucleus |
| Atp Synthase α subunit | Solyc09g056100.1.1 | 8,1 | mtDNA |
| Cytochrome c oxidase subunit 1 | Solyc11g063610.1.1 | 5,9 | Nucleus |
| Monooxygenase FAD-binding protein | Solyc08g063130.2.1 | 4,5 | Nucleus |
| ATP synthase β subunit | Solyc11g039980.1.1 | 3,6 | Nucleus |
| NADH-quinone oxidoreductase subunit D | Solyc01g065780.1.1 | 2,9 | mtDNA |
| ATP synthase α subunit | Solyc03g043610.1.1 | 2,7 | Nucleus |
| Cytochrome c oxidase subunit VC | Solyc01g079420.2.1 | 2,6 | Nucleus |
| NADH dehydrogenase subunit 5 | Solyc00g013140.1.1 | 2,1 | Nucleus |
| Cytochrome c biogenesis | Solyc00g020040.1.1 | 2,0 | Nucleus |
| Formate dehydrogenase | Solyc02g086880.2.1 | 2,0 | Nucleus |
| **Pentatricopeptide** |  |  |  |
| PCMP-H61 | Solyc03g006990.1.1 | 10,7 | Nucleus |
| PCMP-E42 | Solyc01g080190.2.1 | 3,8 | Nucleus |
| PCMP-H26 | Solyc03g115800.1.1 | 3,3 | Nucleus |
| PCMP-H45 | Solyc07g006990.1.1 | 2,5 | Nucleus |
| PCMP-E98 | Solyc11g008970.1.1 | 2,5 | Nucleus |
| PCMP-E101 | Solyc03g118080.2.1 | 2,4 | Nucleus |
| PCMP-H24 | Solyc05g006460.1.1 | 2,3 | Nucleus |
| PCMP-E33 | Solyc04g016540.1.1 | 2,3 | Nucleus |
| PCMP-E86 | Solyc09g007040.1.1 | 2,3 | Nucleus |
| Pentatricopeptide repeat-containing protein | Solyc02g086230.1.1 | 2,2 | Nucleus |
| Pentatricopeptide repeat-containing protein | Solyc01g100340.2.1 | 2,1 | Nucleus |
| Pentatricopeptide repeat-containing protein | Solyc02g085650.2.1 | 2,1 | Nucleus |
| PCMP-E64 | Solyc01g100800.1.1 | 2,1 | Nucleus |
| Pentatricopeptide repeat-containing protein | Solyc07g053380.1.1 | 2,1 | Nucleus |
| Pentatricopeptide repeat-containing protein | Solyc10g081880.1.1 | 2,1 | Nucleus |
| Pentatricopeptide repeat-containing protein | Solyc04g005460.1.1 | 2,1 | Nucleus |
| PCMP-H12 | Solyc10g084540.1.1 | 2,1 | Nucleus |
| PCMP-E91 | Solyc10g007940.1.1 | 2,1 | Nucleus |
| PCMP-H89 | Solyc05g023900.1.1 | 2,0 | Nucleus |
| PCMP-E83 | Solyc01g099000.1.1 | 2,0 | Nucleus |
| Pentatricopeptide repeat-containing protein | Solyc01g088490.2.1 | 2,0 | Nucleus |
| PCMP-E77 | Solyc01g112200.2.1 | 2,0 | Nucleus |
| PCMP-H24 | Solyc02g076700.1.1 | 2,0 | Nucleus |
| **Transcription and Translation** |  |  |  |
| Ribosomal protein S4 | Solyc00g021640.2.1 | 5,9 | Nucleus |
| 50S Ribossomal Protein L20 | Solyc02g069840.2.1 | 4,1 | Nucleus |
| 50S ribosomal protein L2 | Solyc12g035840.1.1 | 4,1 | Nucleus |
| Intron Maturase type II | Solyc11g056270.1.1 | 3,3 | Nucleus |
| Ribosomal protein S3 | Solyc05g045830.1.1 | 2,7 | Nucleus |
| 30S ribosomal protein S13 | Solyc11g056310.1.1 | 2,4 | Nucleus |
| tRNA dimethylallyltransferase | Solyc01g080150.2.1 | 2,4 | Nucleus |
| **Lipid Metabolism** |  |  |  |
| GNS1/SUR4 membrane family protein | Solyc02g089860.1.1 | 15,0 | Nucleus |
| ER glycerol-phosphate acyltransferase | Solyc07g056320.2.1 | 5,0 | Nucleus |
| Enoyl-(Acyl-carrier-protein) reductase II | Solyc01g105370.2.1 | 2,5 | Nucleus |
| Hydroxymethylglutaryl-CoA synthase | Solyc08g007790.2.1 | 2,5 | Nucleus |
| **Aminoacid Metabolism** |  |  |  |
| Proline dehydrogenase | Solyc02g089630.2.1 | 2,7 | Nucleus |
| Lysine ketoglutarate reductase | Solyc02g078820.2.1 | 2,1 | Nucleus |
| **Auxin-induced** |  |  |  |
| ARGOS | Solyc12g096570.1.1 | 3,3 | Nucleus |
| Auxin-induced SAUR-like protein | Solyc03g033590.1.1 | 2,3 | Nucleus |
| **Other function** |  |  |  |
| Cation diffusion facilitator 9 | Solyc12g017350.1.1 | 231,6 | Nucleus |
| Lycopene Beta Cyclase | Solyc06g074240.1.1 | 18,0 | Nucleus |
| Cell wall-associated hydrolase | Solyc11g044610.1.1 | 3,6 | mtDNA |
| DVL1 | Solyc02g065570.1.1 | 3,0 | Nucleus |
| Tetratricopeptide-like protein | Solyc06g065910.2.1 | 2,6 | Nucleus |
| Major facilitator superfamily transporter | Solyc04g073960.2.1 | 2,4 | Nucleus |
| Peroxisomal membrane protein Mpv17/PMP22 | Solyc06g053550.2.1 | 2,4 | Nucleus |
| Extracellular ligand-gated ion channel | Solyc07g064900.2.1 | 2,0 | Nucleus |
| **Function Unknown** |  |  |  |
| Unknown Protein | Solyc08g081850.1.1 | 18,2 | Nucleus |
| Unknown Protein | Solyc05g045730.1.1 | 14,4 | mtDNA |
| Unknown Protein | Solyc11g045440.1.1 | 8,0 | mtDNA |
| Uncharacterized mitochondrial protein | Solyc12g042340.1.1 | 7,2 | Nucleus |
| Uncharacterized mitochondrial protein | Solyc10g048060.1.1 | 6,2 | mtDNA |
| Unknown Protein | Solyc03g058300.1.1 | 5,0 | mtDNA |
| Unknown Protein | Solyc11g030910.1.1 | 3,8 | mtDNA |
| Unknown Protein | Solyc08g036520.2.1 | 3,4 | Nucleus |
| Unknown Protein | Solyc00g014840.1.1 | 3,3 | Nucleus |
| CM0545.320.nc protein | Solyc11g066010.1.1 | 3,0 | Nucleus |
| Unknown protein | Solyc12g036570.1.1 | 2,9 | mtDNA |
| Os10g0422600 protein | Solyc07g042190.2.1 | 2,5 | Nucleus |
| Uncharacterized mitochondrial protein | Solyc03g005630.2.1 | 2,3 | Nucleus |
| Unknown Protein | Solyc03g064040.1.1 | 2,3 | mtDNA |
| Unknown Protein | Solyc08g061450.1.1 | 2,3 | Nucleus |
| Unknown Protein | Solyc02g088130.1.1 | 2,1 | Nucleus |
| Unknown Protein | Solyc05g006610.2.1 | 2,1 | Nucleus |
| Unknown Protein | Solyc03g063790.1.1 | 2,1 | mtDNA |
| Uncharacterized mitochondrial protein | Solyc00g126010.1.1 | 2,0 | Nucleus |
| Unknown Protein | Solyc07g042330.1.1 | 2,0 | Nucleus |
| Unknown Protein | Solyc03g098130.2.1 | 2,0 | Nucleus |
