# Supplementary material for: Overexpression of UCP1 in tobacco induces mitochondrial biogenesis and amplifies a broad stress response
Source: BMC Plant Biol. 2014 May 28;14:144. doi: 10.1186/1471-2229-14-144 (PMC4046140; doi:10.1186/1471-2229-14-144)
Supplement: Additional file 3: Table S2 — Predicted mitochondrial genes upregulated with ≥ 2-fold change in P07 compared with WT. The prediction was based on TargetP. [file 1471-2229-14-144-S3.docx]

**Supplemental Table 2.** Predicted mitochondrial genes upregulated with ≥ 2-fold change in P07 compared with WT. The prediction was based on TargetP.

| **Uniprot description** | ***S. Lycopersicum* ID** | **Fold Change** |
| --- | --- | --- |
| Sister chromatid cohesion 1 protein 1 | Solyc06g074870.1.1 | 16,2 |
| Unknown Protein | Solyc03g083760.1.1 | 15,9 |
| Unknown Protein | Solyc00g005860.1.1 | 6,0 |
| Late embryogenesis abundant protein | Solyc02g062770.1.1 | 5,5 |
| Unknown Protein | Solyc03g058300.1.1 | 5,0 |
| 60S ribosomal protein L33-B | Solyc03g096360.2.1 | 4,7 |
| Unknown Protein | Solyc00g281110.1.1 | 4,6 |
| Wound induced protein | Solyc07g054790.1.1 | 4,6 |
| Helicase-like protein | Solyc10g045660.1.1 | 4,5 |
| NBS-LRR class disease resistance protein | Solyc07g056190.2.1 | 4,2 |
| Ulp1 peptidase-like | Solyc08g016280.1.1 | 4,1 |
| Os11g0198100 protein | Solyc02g078180.2.1 | 4,0 |
| Unknown Protein | Solyc06g074570.1.1 | 4,0 |
| Protein phosphatase 2C | Solyc01g087460.1.1 | 3,9 |
| UDP-glucosyltransferase HvUGT5876 | Solyc04g016200.1.1 | 3,9 |
| Unknown Protein | Solyc11g030910.1.1 | 3,8 |
| Unknown Protein | Solyc06g075120.1.1 | 3,5 |
| Polyprotein | Solyc08g016700.1.1 | 3,5 |
| Pol polyprotein | Solyc00g131710.1.1 | 3,4 |
| Unknown Protein | Solyc07g032350.1.1 | 3,4 |
| Phosphatase 2C family protein | Solyc10g084410.1.1 | 2,9 |
| NADH-quinone oxidoreductase subunit D | Solyc01g065780.1.1 | 2,9 |
| Unknown Protein | Solyc00g020030.1.1 | 2,8 |
| CM0216240nc protein | Solyc02g083120.1.1 | 2,8 |
| Nbs-lrr resistance protein | Solyc07g055380.1.1 | 2,8 |
| Xyloglucan endotransglucosylase/hydrolase 3 | Solyc07g006850.1.1 | 2,7 |
| Unknown Protein (AHRD V1) | Solyc08g079810.2.1 | 2,6 |
| Unknown Protein | Solyc00g010810.1.1 | 2,6 |
| Coiled-coil domain-containing protein 109A | Solyc04g079910.2.1 | 2,5 |
| Sodium/calcium exchanger protein | Solyc07g042000.2.1 | 2,4 |
| Unknown Protein | Solyc04g051390.2.1 | 2,3 |
| Endonuclease/exonuclease/phosphatase | Solyc11g062160.1.1 | 2,3 |
| Lysine ketoglutarate reductase trans-splicing related 1-like | Solyc11g008930.1.1 | 2,3 |
| Ankyrin repeat-containing protein | Solyc02g068670.1.1 | 2,3 |
| Auxin responsive SAUR protein | Solyc07g066560.1.1 | 2,2 |
| Pentatricopeptide repeat-containing protein | Solyc09g005710.1.1 | 2,2 |
| Unknown Protein | Solyc09g098510.2.1 | 2,2 |
| Metallophosphoesterase | Solyc07g005120.2.1 | 2,0 |
| Phosphatase 2C family protein | Solyc01g107310.2.1 | 2,0 |
| Cc-nbs-lrr resistance protein | Solyc05g044490.2.1 | 2,0 |
| Mitochondrial import inner membrane translocase TIM14 | Solyc06g072330.2.1 | 2,0 |
| alpha/beta fold family protein | Solyc11g007030.1.1 | 2,0 |
| Transposase | Solyc02g050190.1.1 | 2,0 |
