# Supplementary material for: Overexpression of UCP1 in tobacco induces mitochondrial biogenesis and amplifies a broad stress response
Source: BMC Plant Biol. 2014 May 28;14:144. doi: 10.1186/1471-2229-14-144 (PMC4046140; doi:10.1186/1471-2229-14-144)
Supplement: Additional file 4: Table S3 — Classification of selected genes involved in different metabolic processes and significantly upregulated in P07 compared with WT. The classification was based on COG. [file 1471-2229-14-144-S4.docx]

**Supplemental Table 3**. Classification of selected genes involved in different metabolic processes and significantly upregulated in P07 compared with WT. The classification was based on COG.

| **UNIPROT description** | ***S. lycopersicum* ID** | **Fold change** | **TAIR ID** | **Reference** |
| --- | --- | --- | --- | --- |
| **Lipid Metabolism** |  |  |  |  |
| AP2-like transcription factor | Solyc08g076380.1.1 | 19,6 | AT3G54320 | Plant J. **60**, 933-47 (2009). |
| Fatty acyl coA reductase | Solyc06g074390.2.1 | 6,9 | AT4G33790 | Plant Physiol. **142**, 866-77 (2006). |
| 4-coumarate CoA ligase-like | Solyc02g088710.2.1 | 6,6 | AT3G21240 | Plant J. **19**, 9-20 (1999). |
| Epoxide hydrolase 3 | Solyc02g078570.2.1 | 5,0 | AT4G02340 | - |
| Patatin-like protein 3 | Solyc02g065090.2.1 | 4,6 | AT4G37060 | - |
| Cytochrome P450 | Solyc10g007860.2.1 | 3,6 | AT1G05160 | Proc. Natl. Acad. Sci. U.S.A. **98**, 2065-70 (2001). |
| Fatty acyl coA reductase | Solyc11g067190.1.1 | 3,6 | AT3G44550 | - |
| Niemann-Pick C1 protein | Solyc04g082490.2.1 | 3,3 | AT4G38350 | - |
| Acyl-protein thioesterase 2 | Solyc08g067160.2.1 | 3,3 | AT5G20060 | - |
| Lipase-like protein | Solyc02g077000.2.1 | 2,7 | AT1G06250 | - |
| Acyl-CoA synthetase II | Solyc02g037490.1.1 | 2,3 | AT5G16370 | Plant Cell. **19**, 3170-93 (2007). |
| Squalene synthase | Solyc10g054130.1.1 | 2,1 | AT4G34640 | Proc. Natl. Acad. Sci. U.S.A. **92**, 2328-32 (1995). |
| Long-chain-fatty-acid--CoA ligase 4 | Solyc12g009040.1.1 | 2,1 | AT3G06860 | Biochem. Soc. Trans. **28,** 95-9 (2000). |
| Acetyl-CoA C-acetyltransferase | Solyc07g045350.2.1 | 2,0 | AT5G47720 | - |
| Patatin-like protein 3 | Solyc05g056030.2.1 | 2,0 | AT2G39220 | - |
| Oxysterol-binding protein | Solyc11g013190.1.1 | 2,0 | AT4G08180 | Trends Plant Sci. **9**, 378-84 (2004). |
| **Calcium Signaling and Transportation** |  |  |  |  |
| Calcium-dependent protein kinase 1 | Solyc01g096820.2.1 | 4,3 | AT5G12180 | Plant J. **59**, 528-39 (2009). |
| Sodium/calcium exchanger protein | Solyc07g006370.1.1 | 3,2 | AT5G17850 | Plant Physiol. **126**, 1646-67 (2001). |
| Calcium-transporting ATPase 1 | Solyc09g082890.1.1 | 2,9 | AT3G22910 | J. Biol. Chem. **277**, 9840-52 (2002). |
| Sodium/calcium exchanger protein | Solyc07g042000.2.1 | 2,4 | AT3G14070 | Plant Physiol. **148**, 1474-86 (2008). |
| Calcium-transporting ATPase 1 | Solyc02g090560.2.1 | 2,3 | AT4G29900 | Plant Physiol. **159**, 798-809 (2012) |
| Calcineurin B-like protein | Solyc08g077770.2.1 | 2,3 | AT4G17615 | Plant Cell. **15**, 1833-45 (2003). |
| C2 domain-containing protein | Solyc07g048040.1.1 | 2,3 | AT5G55530 | - |
| Calcium-binding EF hand protein | Solyc10g061870.1.1 | 2,2 | AT1G64850 | - |
| Calcium-transporting ATPase 1 | Solyc04g016260.2.1 | 2,1 | AT2G41560 | Plant Physiol. **124**, 1814-27 (2000). |
| Calcium-responsive transactivator | Solyc04g009820.2.1 | 2,1 | AT3G57330 | Plant Physiol. **154**, 1158-71 (2010). |
| Calcium/proton exchanger protein | Solyc07g056110.2.1 | 1,8 | AT3G13320 | FEBS Lett. **579**, 2648-56 (2005). |
| Calcium dependent protein kinase | Solyc01g096350.2.1 | 1,8 | AT2G46700 | Biochem. Biophys. Res. Commun. **342**, 119-26 (2006). |
| Calcium-dependent protein kinase 3 | Solyc01g112250.2.1 | 1,6 | AT4G35310 | - |
| Calcium-dependent protein kinase | Solyc02g032820.2.1 | 1,6 | AT4G04720 | Mol Plant. **4**, 83-96 (2011). |
| C2 domain-containing protein | Solyc07g008070.1.1 | 1,6 | AT2G33320 | - |
| Calcium-dependent protein kinase 8 | Solyc11g065660.1.1 | 1,6 | AT5G12480 | - |
| **MPTP Opening** |  |  |  |  |
| VDAC Protein | Solyc07g008350.2.1 | 1,4 | AT3G01280 | Plant Mol Biol. **78**, 431-46 (2012). |
| VDAC Protein | Solyc02g092440.2.1 | 1,4 | AT5G15090 | Plant Mol Biol. **78**, 431-46 (2012). |
| Mitochondrial ornithine transporter | Solyc10g009090.2.1 | 1,9 | AT2G33820 | Plant J. **33,** 1027-35 (2003). |
| Mitochondrial carrier protein | Solyc09g011360.2.1 | 1,8 | AT3G53940 | - |
| Mitochondrial carrier protein | Solyc06g082290.2.1 | 1,8 | AT2G30160 | - |
| Mitochondrial carrier protein | Solyc01g095510.2.1 | 1,6 | AT4G27940 | Planta. **226**, 1031-9 (2007). |
| Mitochondrial carrier protein | Solyc12g008770.1.1 | 1,5 | AT4G28390 | Eur. J. Biochem. **269**, 3172-81 (2002). |
| Mitochondrial ADP/ATP carrier | Solyc07g053830.2.1 | 1,4 | AT5G13490 | Eur. J. Biochem. **269**, 3172-81 (2002). |
| ADPATP carrier protein 1 | Solyc08g063030.2.1 | 1,4 | AT4G01100 | Plant Physiol. **148**, 1797-808 (2008). |
| **Mitochondrion-Microtubule association** |  |  |  |  |
| Misato homolog 1 | Solyc01g100190.2.1 | 1.4 | AT4G37190 | Exp Cell Res. **313**, 1393-404 (2007). |
| **Fission Proteins** |  |  |  |  |
| FIS1a | Solyc11g065100.1.1 | 1,4 | AT3G57090 | Mol Plant. **1**, 1036-47 (2008). |
| FIS1b | Solyc12g099770.1.1 | 1,3 | AT5G12390 | Mol Plant. **1**, 1036-47 (2008). |
| Dynamin-2A | Solyc01g103120.2.1 | 1,7 | AT1G59610 | Proc. Natl. Acad. Sci. U.S.A. **107**, 6094-9 (2010). |
| Dynamin-1A | Solyc01g095970.2.1 | 1,4 | AT5G42080 | Plant Cell. **15**, 899-913 (2003). |
| **WRKY Transcription Factors** |  |  |  |  |
| WRKY transcription factor 73 | Solyc06g070990.2.1 | 19,9 | AT5G15130 | - |
| WRKY transcription factor 6 | Solyc05g050340.2.1 | 2,8 | AT4G11070 | - |
| WRKY transcription factor 23 | Solyc08g081610.2.1 | 2,3 | AT4G01250 | Mol. Cells. **31**, 303-13 (2011). |
| WRKY transcription factor 5 | Solyc07g055280.2.1 | 2,3 | AT1G29280 | - |
| WRKY transcription factor 5 | Solyc02g072190.2.1 | 2,0 | AT3G58710 | - |
| WRKY transcription factor | Solyc07g051840.2.1 | 1,9 | AT1G62300 | Plant Cell. **21**, 3554-66 (2009). |
| Transcription factor WRKY | Solyc02g080890.2.1 | 1,8 | AT4G04450 | - |
| WRKY family transcription factor | Solyc04g051540.2.1 | 1,7 | AT4G39410 | - |
| DNA-binding WRKY VQ | Solyc02g078030.1.1 | 1,6 | AT1G28280 | Plant Physiol. **159**, 810-25 (2012). |
| WRKY transcription factor 32 | Solyc01g104550.2.1 | 1,5 | AT1G68150 | - |
| **TCP Transcription Factors** |  |  |  |  |
| TCP family transcription factor | Solyc08g080150.1.1 | 3,1 | AT2G45680 | - |
| Transcription factor TCP | Solyc06g069460.1.1 | 1,9 | AT3G02150 | Plant Cell. **20**, 2293-306 (2008). |
| TCP13 | Solyc03g115010.1.1 | 1,6 | AT1G30210 | Plant Cell. **17**, 2693-704 (2005). |
| CYCLOIDEA | Solyc10g008780.1.1 | 1,5 | AT2G31070 | Plant J. **71**, 99-107 (2012). |
| TCP3 | Solyc07g053410.2.1 | 1,5 | AT1G53230 | - |
| **bZIP Transcription Factors** |  |  |  |  |
| BZIP transcription factor | Solyc01g079480.2.1 | 1,4 | AT3G62420 | Plant Physiol. **150**, 84-95 (2009). |
| **Import Machinery** |  |  |  |  |
| TXR1 | Solyc08g006120.2.1 | 1,7 | AT3G59280 | - |
| PAM16 | Solyc06g068820.2.1 | 1,5 | AT5G61880 | - |
| TIM23-2 | Solyc03g121860.1.1 | 1,4 | AT1G72750 | Plant Cell. **24**, 2675-95 (2012). |
| OXA1 | Solyc08g008320.2.1 | 1,4 | AT5G62050 | Plant Cell Physiol. **41**, 1157-63 (2000). |
| Metaxin 2 | Solyc07g009320.2.1 | 1,4 | AT2G19080 | Plant Cell. **19**, 3739-59 (2007). |
