# Supplementary material for: Overexpression of UCP1 in tobacco induces mitochondrial biogenesis and amplifies a broad stress response
Source: BMC Plant Biol. 2014 May 28;14:144. doi: 10.1186/1471-2229-14-144 (PMC4046140; doi:10.1186/1471-2229-14-144)
Supplement: Additional file 5: Table S4 — Abiotic stress-responsive genes upregulated in P07 compared with WT. The classification was based on STIFDB and shows the most upregulated genes involved in different types of abiotic stresses. [file 1471-2229-14-144-S5.docx]

**Supplemental Table 4.** Abiotic stress-responsive genes upregulated in P07 compared with WT. The classification was based on STIFDB and shows the most upregulated genes involved in different types of abiotic stresses.

| **Stress/UNIPROT Description** | ***S. Lycopersicum* ID** | **Fold change** | **TAIR ID** | **Reference** |
| --- | --- | --- | --- | --- |
| **Heat** |  |  |  |  |
| Heat shock protein | Solyc01g099660.2.1 | 6,6 | AT5G42020 | Plant Cell Physiol. **37**:862-5 (1996). |
| Heat shock protein | Solyc11g066060.1.1 | 2,7 | AT3G12580 | Plant Physiol. **126**, 789-800 (2001). |
| Heat stress transcription factor A3 | Solyc08g062960.2.1 | 2,3 | AT5G03720 | Plant J. **53**, 264-74 (2008). |
| Heat shock protein | Solyc11g066100.1.1 | 2,2 | AT3G12580 | Plant Physiol. **126**,789-800 (2001). |
| Heat shock protein | Solyc03g082920.2.1 | 2,0 | AT1G09080 | Plant Cell Physiol. **37**, 862-5 (1996). |
| Chaperone protein dnaJ 2 | Solyc02g077670.2.1 | 1,9 | AT2G20560 | - |
| Chaperone protein DnaJ | Solyc02g014860.2.1 | 1,8 | AT4G39960 | - |
| Heat stress transcription factor | Solyc03g026020.2.1 | 1,7 | AT4G36990 | Mol. Gen. Genet. **258**, 269-78 (1998). |
| Cytochrome P450 | Solyc07g062500.2.1 | 1,7 | AT3G14620 | - |
| Alpha-amylase | Solyc03g095710.2.1 | 1,7 | AT4G25000 | Plant Cell Environ. **30**, 388-98 (2007). |
| **Osmotic** |  |  |  |  |
| GID1-like gibberellin receptor | Solyc06g008870.2.1 | 3,6 | AT3G63010 | - |
| Protein kinase 2 | Solyc03g095510.2.1 | 2,1 | AT3G08720 | Plant Cell. **18**, 477-90 (2006). |
| Calcium-transporting ATPase 1 | Solyc04g016260.2.1 | 2,1 | AT2G41560 | Plant Physiol. **124**, 1814-27 (2000). |
| U-box domain-containing protein | Solyc05g056500.1.1 | 1,6 | AT3G19380 | - |
| Chorismate mutase 2 | Solyc11g017240.1.1 | 1,5 | AT5G10870 | - |
| Folate/biopterin transporter | Solyc12g005140.1.1 | 1,5 | AT5G54860 | - |
| **Cold** |  |  |  |  |
| Unknown Protein | Solyc10g050220.1.1 | 11,7 | AT4G33980 | - |
| TPX-2 | Solyc07g052530.2.1 | 8,6 | At1g05260 | Plant J. **32**, 13-24 (2002). |
| ABC transporter G family member 37 | Solyc01g101070.2.1 | 4,2 | AT3G53480 | - |
| Pyruvate decarboxylase 1 | Solyc09g005110.2.1 | 4,0 | AT4G33070 | - |
| Pyruvate decarboxylase | Solyc10g076510.1.1 | 3,9 | AT4G33070 | - |
| Receptor-like protein kinase | Solyc04g006930.2.1 | 3,7 | AT4G18670 | - |
| UDP-glucosyltransferase | Solyc00g227860.1.1 | 3,7 | AT2G22590 | - |
| Mate efflux family protein | Solyc11g010380.1.1 | 3,6 | AT5G65380 | [-](http://www.ncbi.nlm.nih.gov/pubmed/19816401) |
| TCP family transcription factor | Solyc08g080150.1.1 | 3,1 | AT2G45680 | - |
| Serpin | Solyc04g079470.2.1 | 3,1 | AT1G47710 | J. Mol. Biol. **364**, 625-36 (2006). |
| CONSTANS-like zinc finger protein | Solyc02g079430.2.1 | 3,0 | AT4G27310 | - |
| UDP-glucosyltransferase | Solyc12g096080.1.1 | 3,0 | AT4G34135 | - |
| Multidrug resistance protein mdtK | Solyc04g074840.2.1 | 2,9 | AT5G65380 | - |
| Omega-6 fatty acid desaturase | Solyc12g044950.1.1 | 2,7 | AT3G12120 | Plant Cell Physiol. **48**, 856-65 (2007). |
| Beta-1 3-glucanase | Solyc04g016470.2.1 | 2,1 | AT3G57260 | J. Exp. Bot. **57**,1537-46 (2006). |
| **Drought** |  |  |  |  |
| Mitochondrial 2-oxoglutarate/malate carrier | Solyc05g051400.2.1 | 13,0 | AT4G24570 | - |
| ABC transporter G family member 22 | Solyc04g070970.2.1 | 8,8 | AT5G06530 | Mol. Cell Proteomics. **6**, 1198-214 (2007). |
| Neutral invertase like protein | Solyc01g111100.2.1 | 4,6 | AT4G34860 | - |
| Timing of CAB expression-like | Solyc03g115770.2.1 | 3,5 | AT5G61380 | EMBO J.**28**,3745-57 (2009). |
| F-box family protein | Solyc12g006130.1.1 | 3,3 | AT3G07870 | - |
| Acyl-protein thioesterase 2 | Solyc08g067160.2.1 | 3,3 | AT5G20060 | - |
| Kinase family protein | Solyc02g091170.2.1 | 3,2 | AT4G18950 | - |
| Hydroxycinnamoyl CoA | Solyc07g005760.2.1 | 3,0 | AT5G48930 | - |
| Zinc finger A20 and AN1 | Solyc01g086970.2.1 | 3,0 | AT3G12630 | - |
| N-acetyltransferase | Solyc08g068780.1.1 | 2,4 | AT2G39030 | - |
| Homeobox leucine zipper protein | Solyc03g082550.2.1 | 2,3 | AT2G46680 | Plant J. **10**, 375-81 (1996). |
| Zinc finger A20 and AN1 | Solyc10g083460.1.1 | 2,2 | AT3G52800 | - |
| F-box family protein | Solyc09g072680.1.1 | 2,2 | AT3G07870 | - |
| CHY zinc finger family protein expressed | Solyc08g067960.2.1 | 2,2 | AT5G25560 | - |
| Prephenate dehydratase | Solyc06g074530.1.1 | 2,1 | AT1G08250 | - |
| **NaCl** |  |  |  |  |
| FAD-binding domain-containing protein | Solyc02g070070.1.1 | 7,7 | AT1G30760 | - |
| Galactinol synthase | Solyc02g084980.2.1 | 6,0 | AT2G47180 | Plant Physiol. **147**,1251-63 (2008). |
| Ammonium transporter | Solyc04g050440.2.1 | 5,7 | AT1G64780 | - |
| Epoxide hydrolase 3 | Solyc02g078570.2.1 | 5,0 | AT3G51000 | - |
| Chaperone protein dnaj | Solyc11g071830.1.1 | 4,0 | AT3G44110 | - |
| Ninja-family protein 3 | Solyc02g088910.2.1 | 3,5 | AT3G29575 | - |
| Glutathione transferase | Solyc08g062570.1.1 | 3,4 | AT1G74590 | - |
| Protein kinase | Solyc04g064590.1.1 | 3,3 | AT1G53570 | - |
| Glutathione S-transferase 12 | Solyc03g116130.1.1 | 3,1 | AT1G74590 | - |
| Hydroxycinnamoyl transferase | Solyc05g052680.1.1 | 3,1 | AT2G39980 | - |
| N-hydroxycinnamoyl/benzoyltransferase | Solyc05g052670.1.1 | 2,9 | AT2G39980 | - |
| Auxin responsive protein | Solyc01g097290.2.1 | 2,8 | AT3G04730 | - |
| Unknown Protein | Solyc12g087960.1.1 | 2,8 | AT4G38060 | - |
| CBL-interacting protein kinase 7 | Solyc12g010130.1.1 | 2,8 | AT4G30960 | Plant J. **58**, 778-90 (2009). |
| Wound-induced protein 1 | Solyc09g014940.1.1 | 2,6 | AT5G01740 | - |
| Glucose transporter member 5 | Solyc03g093400.2.1 | 2,5 | AT5G61520 | - |
| Wound-induced protein 1 | Solyc06g050760.1.1 | 2,5 | AT3G10985 | - |
| Ethylene responsive transcription factor | Solyc12g056980.1.1 | 2,4 | AT1G78080 | FEBS J. **276**,6624-35 (2009). |
| NF-X1 finger transcription factor | Solyc03g118420.2.1 | 2,4 | AT1G10170 | FEBS Lett. **580**, 4851-6 (2006). |
| **Oxidative** |  |  |  |  |
| Cevi-16 | Solyc02g064970.2.1 | 19,9 | AT5G39580 | - |
| Class I heat shock protein | Solyc10g086680.1.1 | 8,4 | AT1G07400 | Plant J. **41**,212-20 (2005). |
| Blue copper protein | Solyc07g008130.2.1 | 7,5 | AT5G20230 | Plant Physiol. **122**, 657-65 (2000). |
| PO2 | Solyc10g084240.1.1 | 3,1 | AT2G37130 | - |
| ClpB chaperone | Solyc03g115230.2.1 | 3,0 | AT1G74310 | Plant J. **48**, 535-47 (2006). |
| Cytochrome P450 | Solyc02g084570.2.1 | 2,7 | AT4G36220 | Plant Physiol. **109**, 1159-66 (1995). |
| Proline dehydrogenase | Solyc02g089630.2.1 | 2,5 | AT3G30775 | Plant Physiol. **116**, 409-18 (1998). |
| POD-5 | Solyc03g044100.2.1 | 2,3 | AT3G49960 | - |
| GH3 family protein | Solyc10g011660.2.1 | 2,3 | AT2G46370 | Plant Physiol. **134**, 1536-45 (2004). |
| FBP-5 | Solyc11g072920.1.1 | 2,2 | AT5G06720 | - |
| Superoxide dismutase | Solyc02g082590.2.1 | 1,5 | AT1G08830 | Plant Cell. **18**, 2051-65 (2006). |
| Glutathione peroxidase | Solyc12g056240.1.1 | 1,4 | AT1G63460 | - |
| Mitochondrial carrier protein | Solyc01g095510.2.1 | 1,6 | AT4G27940 | Planta. **226**, 1031-9 (2007). |
| **Cadmium** |  |  |  |  |
| Pyruvate kinase | Solyc09g008840.2.1 | 2,8 | AT3G52990 | Proteomics. **6**, 2180-98 (2006). |
| Chitinase | Solyc10g055800.1.1 | 3,7 | AT3G12500 | Proteomics. **6**, 2180-98 (2006). |
| Multidrug resistance protein 2 | Solyc10g055770.1.1 | 2,2 | AT3G12500 | Proteomics. **6**, 2180-98 (2006). |
| Enoyl-(Acyl-carrier-protein) reductase II | Solyc01g105370.2.1 | 2,5 | AT5G64250 | J. Exp. Bot.**57**, 4003-13 (2006). |
| **Other** |  |  |  |  |
| WRKY transcription factor | Solyc07g051840.2.1 | 1,9 | AT1G62300 | Plant Cell.**21**, 3554-66 (2009) |
| NAC domain transcription factor | Solyc04g078670.2.1 | 1,8 | AT5G63790 | Plant Physiol. **149**, 1724-38 (2009). |
| ELF4-like protein | Solyc06g051680.1.1 | 5,5 | AT2G40080 | Plant Physiol.**133**, 1530-8 (2003). |
| Homeobox-leucine zipper protein | Solyc08g078300.2.1 | 4,4 | AT4G16780 | Plant J. **4**, 469-79 (1993). |
|  |  |  |  |  |
