# Supplementary material for: Overexpression of UCP1 in tobacco induces mitochondrial biogenesis and amplifies a broad stress response
Source: BMC Plant Biol. 2014 May 28;14:144. doi: 10.1186/1471-2229-14-144 (PMC4046140; doi:10.1186/1471-2229-14-144)
Supplement: Additional file 6: Table S5 — Summary of RNASeq data filtered and mapped with Bowtie. [file 1471-2229-14-144-S6.docx]

**Supplemental Table 5**. Summary of RNASeq data filtered and mapped with Bowtie.

| **Library** | **Sequenced reads** | **Filtered reads** | **Mapped reads** | **% of mapped reads** |
| --- | --- | --- | --- | --- |
| P07P1F3 | 28.427.445 | 27.905.560 | 20.912.405 | 74,94 |
| P07P2F3 | 100.466.818 | 90.289.253 | 66.222.859 | 73,35 |
| P07P3F3 | 67.479.470 | 65.760.027 | 48.887.230 | 74,34 |
| P07P4F3 | 57.941.595 | 56.154.368 | 41.363.058 | 73,66 |
| WTP1F3 | 115.484.011 | 113.267.169 | 79.782.022 | 70,44 |
| WTP2F3 | 61.768.888 | 55.603.556 | 39.791.178 | 71,56 |
| WTP3F3 | 85.125.227 | 83.066.501 | 59.319.951 | 71,41 |
| WTP4F3 | 52.352.176 | 50.748.225 | 36.352.829 | 71,63 |
| **Total** | **569.045.630** | **542.794.659** | **392.631.532** | **72,33** |
